# ML-automated microfluidic circuit design

**DOI:** 10.1126/sciadv.aea7598

**Published:** 2026-01-28

**Authors:** Mehmet Tugrul Birtek, Vural Aktas, Bora Aktas, Ahmed Choukri Abdullah, Aydogan Ozcan, Savas Tasoglu

**Affiliations:** ^1^ Department of Biomedical Sciences and Engineering, Koç University, Sariyer, Istanbul, Turkey 34450.; ^2^ Department of Mechanical Engineering, Koç University, Sariyer, Istanbul, Turkey 34450.; ^3^ Department of Industrial Engineering, Koç University, Sariyer, Istanbul, Turkey 34450.; ^4^ Department of Electrical and Computer Engineering, University of California, Los Angeles, CA 90095, USA.; ^5^ Department of Bioengineering, University of California, Los Angeles, CA 90095, USA.; ^6^ California NanoSystems Institute (CNSI), University of California, Los Angeles, CA 90095, USA.; ^7^ Department of Surgery, David Geffen School of Medicine, University of California, Los Angeles, CA, USA.; ^8^ Koç University Translational Medicine Research Center (KUTTAM), Koç University, Sariyer, Istanbul, Turkey 34450.; ^9^ Koç University Is Bank Artificial Intelligence Lab (KUIS AILab), Koç University, Sariyer, Istanbul, Turkey 34450.; ^10^ Koç University Arçelik Research Center for Creative Industries (KUAR), Koç University, Sariyer, Istanbul, Turkey 34450.; ^11^ Boğaziçi Institute of Biomedical Engineering, Boğaziçi University, Çengelköy, Istanbul, Turkey 34684.

## Abstract

Microfluidics enable high-precision and cost-effective processing of biological and chemical substances. However, designing and fabricating microfluidic chips typically requires substantial expertise and numerous design iterations, posing considerable barriers to entry for nonexperts. We introduce μFluidicGenius (μFG), an open-access, machine learning (ML)–augmented design tool that enables nonexpert users to rapidly create functional microfluidic circuits. Users simply define the spatial placement of reservoirs, specify the channel connections between them, and assign desired flow rates through this layout. Leveraging a hybrid algorithmic framework that integrates ML models with mathematical modeling, μFG automatically generates spatially coded maze structures that implement the precise fluidic resistances needed to meet the target flow distribution. These resistive elements are optimized to fit within the available geometry and can reproduce complex flow profiles, such as physiologically relevant flow rates in multi-organ-on-chip platforms. The resulting microfluidic designs are directly exportable for three-dimensional printing. Experimental validation demonstrates that μFG-generated circuits reproduce target flow distributions with 90% accuracy. By streamlining and automating microfluidic circuit creation, μFG not only lowers the barrier to entry for nonexperts but also showcases a principled and efficient application of ML to fluidic system design, enabling rapid and customizable development of complex microfluidic architectures.

## Teaser

A machine learning tool automatically designs functional microfluidic chips from simple user inputs.

## INTRODUCTION

Fully integrated, automatic liquid processing has been an enduring ambition in microfluidics (*1*, *2*). Microfluidic channel sizes vary from a few to hundreds of microns (*3*–*5*), and they enable manipulation of fluids and materials at this scale (*6*, *7*). Microfluidic chips consist of a network of microchannels that serve as a fluidic circuit connected to the environment (*8*, *9*). Fluids can be operated to perform directing, mixing, analysis, and reactions in biomedical (*10*–*14*), chemical (*15*, *16*), and environmental (*17*, *18*) applications. The cutting-edge microfluidic innovation is used within biomedical research. Organ-on-chip technology offers a platform for studying organ-level functions, drug responses, and disease mechanisms in vitro (*19*–*21*). Rapid, high-throughput sorting and separation of target biological particles from minimized fluid samples is accomplished with complex microfluidic structures (*22*–*28*). Microfluidic contact lenses empower diagnosis and treatment of scleral diseases (*29*, *30*). Implementation of state-of-the-art biological techniques within microfluidic circuits provides precise point-of-care diagnostics (*31*–*33*). Furthermore, cost-effective and quantitative examination of body fluids such as urine and sperm facilitates precise health tracking (*34*–*36*).

Most of these applications necessitate accurate control of flow parameters such as volume, flow rate, and pressure (*37*, *38*). Optimization of such control systems is mostly achieved by rigorous experimental research. Alternatively, computational fluid dynamics (*39*–*42*) or finite element analysis (*43*–*45*) can be used to predict performance of microfluidic structures numerically. Yet, they require expertise to implement and demand huge computational resources (*46*). Machine learning (ML) techniques analyze vast sets of intricate data swiftly, offering heightened comprehension and accurate forecasts within shorter time frames (*47*). Integrating ML analysis methods within microfluidic chips increases the effectiveness of biological and chemical micro-systems (*48*). Furthermore, such algorithms are used to excel design and the manufacturing of microfluidic channels (*49*). The automation of designing droplet generators has advanced considerably through the implementation of ML tools (*50*–*52*). Likewise, precise flow manipulation (*53*) and micromixing (*54*, *55*) have been achieved by optimizing microchannel geometry using ML techniques.

**Fig. 1.**
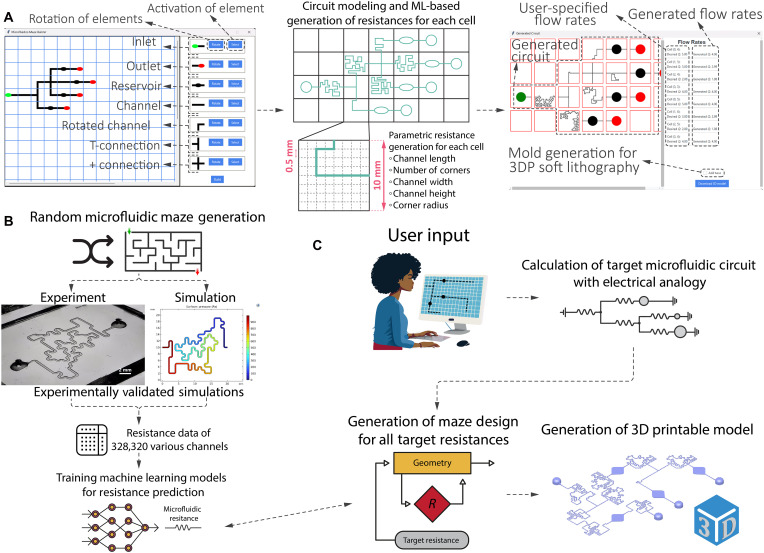
Outline of the μFG. (**A**) Interface of the μFG. The user inputs multiple reservoirs and connections on the interface and specifies flow rates for each reservoir and channel. The software calculates the circuit and designs microfluidic mazes with specific resistances. Last, a 3D printable file is given to the user by the algorithm. 3DP, 3D printed. (**B**) Description of data generation method. The method of simulation was experimentally validated for six different geometries. Random mazes were simulated for generating a dataset containing 328,320 different geometries. The ML algorithm was trained on this dataset to predict microfluidic resistance. (**C**) The μFG uses electrical circuit analogy to calculate resistance requirement for each channel in the design. Generative algorithm then devises geometries to meet the required resistance values for the circuit. A 3D printable .stl file was subsequently exported to the user.

The design and manufacturing of precisely controlled microfluidic structures, analogous to electrical circuits, remains an enduring ambition (*56*–*58*). However, progress was hindered by manufacturing limitations and computational complexity. Here, we introduce an ML-augmented, high-precision microfluidics tool for automated designing of microfluidic circuits. The μFluidicGenius (μFG) interface allows users to drag-and-drop reservoirs and channels within a predefined space and specify the desired flow rates through each reservoir. The tool was trained on an extensive, experimentally validated simulation dataset. Using this training data, the generative ML tool tailors a maze-like geometry within a defined area to achieve precise microfluidic resistance values. The electrical circuit analogy was used in conjunction with the ML-enabled resistance prediction module to generate specified microfluidic circuits. After making these calculations, the μFG exports a three-dimensional (3D) printable model of the microfluidic chip with the desired properties as a final product. The practicality of the software was validated by 3D printing three different chips designed via μFG. An outline of this study is provided in Fig. 1.

## RESULTS

### Generation of comprehensive dataset

Microfluidic resistance, defined as the flow response of a channel under a given pressure difference, is governed by the channel’s geometry [width (*w*), height (*h*), and length (*L*)] and fluidic parameters. Achieving a broad range of resistance values typically requires long, narrow channels that occupy large chip areas or demand additional external components. To overcome this limitation, we designed maze-like channel layouts that compactly realize the desired hydraulic resistance within a fixed footprint. These geometries provide efficient access to both low- and high-resistance regimes while minimizing device area and eliminating the need for auxiliary extensions, making them particularly advantageous when space and material resources are constrained.

**Fig. 2.**
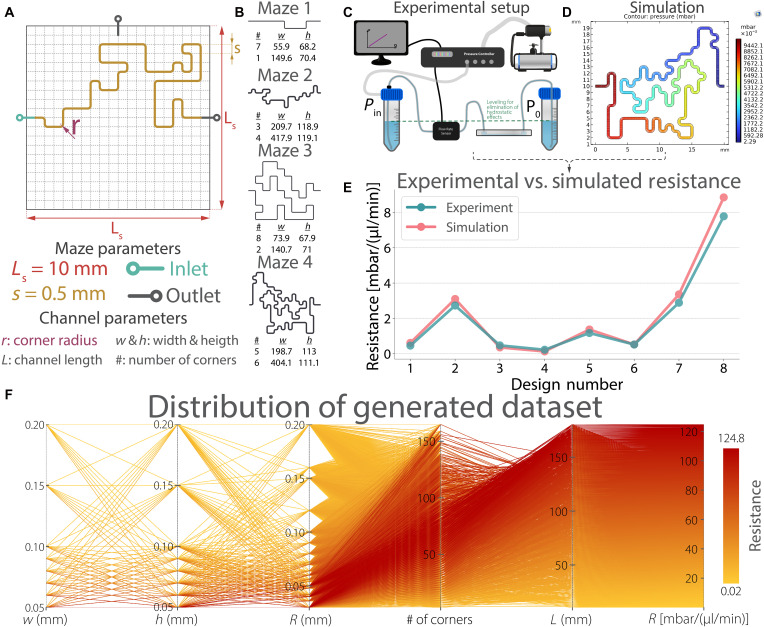
Schematic of maze unit cell design and microfluidic channel parameters, method of data generation, and the distribution of the generated dataset. (**A**) The edge length of each unit cell was fixed at 10 mm, while the step size of the random mazes was set to 0.5 mm, considering the manufacturing limitations of 3D printing. The inlet to the unit cell was fixed at the edge shown, while the outlet was varied between two different edges. Mazes were rotated when inlets and outlets needed to be placed on different edges of the unit cell. The microfluidic parameters (*w*, *h*, and *R*) were applied to coordinates generated by the maze algorithm. (**B**) List of the microfluidic mazes used in experimental validation of simulations. Dimensions listed are in micrometers. Eight channels were manufactured and characterized using the experimental setup described in (**C**). (**D**) Simulation interface showing the pressure distribution along a channel. Resistance was calculated by dividing the pressure drop between the inlet and outlet by the average flow rate. The eight designs were performed with both experimental setup and the simulation method. (**E**) Their comparison plot, showing a strong correlation [$R^{2}=0.965$ and average absolute deviation of 0.31 mbar/(μl/min)], proving the feasibility of the simulation method. (**F**) Distribution of the generated dataset containing 382,320 unique resistance values. Line colors indicate hydraulic resistance (*R*), ranging from yellow for low to red for high values. The parameters *w*, *h*, *r*, and *L* denote channel width, height, corner radius, and total channel length, respectively. The dataset spans ~0 to 124 mbar/(μl/min), with resistance values mainly distributed according to variations in *L*. The parameters *w* and *h* were discretely sampled at consistent integer values, while the number of corners introduced the second-largest variation across maze geometries. The corner radius (*r*) further contributed minor additional variability to the overall resistance distribution.

To systematically explore how geometric design influences this resistance in single-phase flows, we developed a Python-based algorithm to create 1710 random microfluidic mazes within a 10 mm–by–10 mm square unit cell, ensuring both compactness and a wide range of channel lengths (from 10 to 180.5 mm). The constraints of these maze pathways were determined by several design parameters as illustrated in Fig. 2A. Each maze was generated by connecting step segments (*S*) of 0.5 mm, with the inlet placed on the “west” edge and the outlet on either the “north” or “east” edge. These mazes were then rotated as needed to achieve other inlet-outlet configurations, a flexibility that facilitated serial integration of multiple mazes.

Next, each maze pathway was converted to microfluidic channels by defining rectangular cross sections (*w* by *h*) along the coordinates of mazes. To validate the simulation approach, we fabricated eight randomly selected mazes and experimentally measured their hydraulic resistances (Fig. 2, B to D). Simulation results demonstrated strong correlation with experimental data [coefficient of determination ($R^{2}$) = 0.965], with an average absolute deviation of 0.31 mbar/(μl/min) (Fig. 2E). Notably, higher percentage errors were observed in low-resistance channels where minor absolute differences result in disproportionately large relative deviations. This highlights the importance of absolute error evaluation, particularly in the low-resistance regime. Details of the validation procedure are explained in section S3.

Building on this validation, we performed a thorough parametric study using COMSOL Multiphysics. A total of 192 unique combinations of *w*, *h*, and *r* across all 1710 mazes (Table 1). Simulations lasted for 30 days and yielded a dataset of 328,320 distinct designs, covering resistance values from 0.02 to 124.80 mbar/(μl/min) (Fig. 2F). Each simulated design was annotated with key geometric parameters, such as total channel length and the number of corners, providing a comprehensive dataset for exploring how microfluidic maze geometry dictates microfluidic resistance.

**Table 1.** Parameter ranges used for data generation. Values of *L* and number of corners represent the minimum and maximum observed across 1710 randomly generated channel paths. The parameters *w* and *h* were systematically swept over the listed discrete values, while *r* was varied across 20 values. This process resulted in a total dataset of 328,320 entries.

Maze designs		Channel width (*w*; μm)	Channel height (*h*; μm)	Corner radius (*r*; μm)
1710		50, 60, 70, 80, 90, 100, 150, 200	50, 60, 70, 80, 90, 100, 150, 200	0, 30, 36, 40, 42, 48, 50, 54, 56, 60, 64, 70, 72, 80, 90, 100, 120, 150, 160, 200
**Length (*L*) variation (mm)**	**Number of corners variation**			
Between 10 and 180.5 with a step size of 0.5	Between 0 and 165 with a step size of 1			

### Resistance prediction with ML

An instance-based ML model was opted to quickly and precisely estimate the resistance of a given microfluidic maze. Resistance was identified as the ultimate objective, and the model was required to work seamlessly with the mathematical framework developed for generating target circuits, thereby ensuring that the designed channels remained robust and 3D printable.

**Fig. 3.**
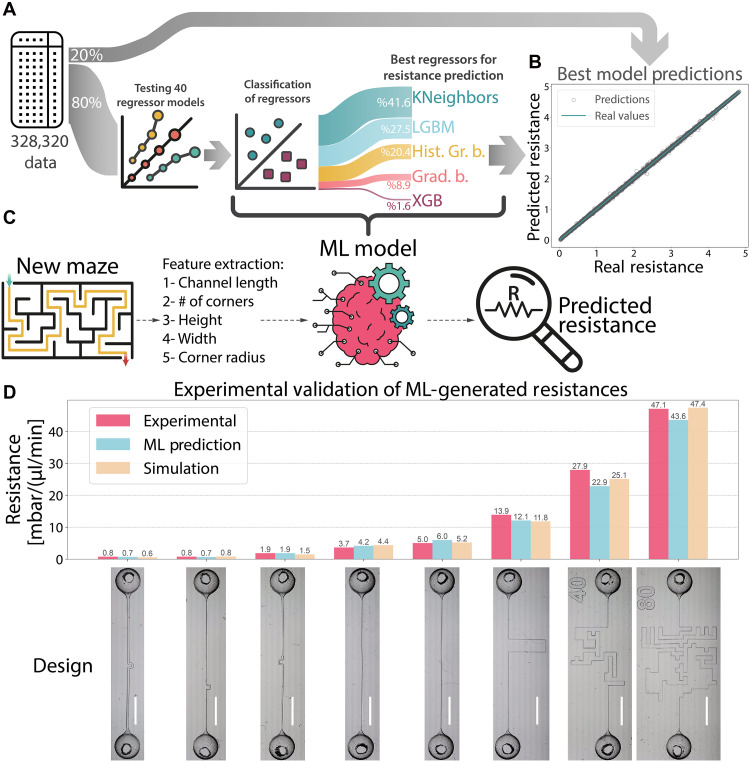
ML framework for microfluidic resistance prediction and experimental validation of μFG-generated resistances. (**A**) Overview of the two-step ML pipeline. The dataset was split into 80% training and 20% testing subsets. First, 27 regressors from the LazyPredict library were trained on the dataset. The top five performing regressors were selected on the basis of accuracy, and a classification model was trained to assign any new input to the most suitable regressor. This classification realized the final hybrid model, which dynamically selected the best regressor for any input. Percentages shown indicate the proportion of data assigned to each regressor. KNeighbors, K nearest neighbors; LGBM, light gradient boosting machine; His. Gr. b., histogram-based gradient boosting; Grad. b., gradient boosting; XGB, extreme gradient boosting. (**B**) Performance of the two-step model on the untouched 20% test set, achieving an *R*^2^ value of 0.999. (**C**) Prediction workflow for unknown maze geometries. When a channel design is provided, the model extracts relevant features, selects an optimal regressor, and predicts the corresponding resistance. This ML engine functions in parallel with μFG’s generative design algorithm. (**D**) Experimental validation of μFG-generated microfluidic resistances. The fabricated channels shown from left to right were designed to yield resistances of 0.5, 1, 2, 4, 8, 10, 20, and 40 mbar/(μl/min). Due to 3D printing–induced dimensional variations, the experimental resistances (shown in pink) deviated slightly from their design targets. The ML model was then used to predict resistances on the basis of the measured channel dimensions, with results shown in blue. COMSOL simulation results using the same measured geometries are also shown for comparison (yellow bars). Scale bars, 5 mm.

The open-source LazyPredict library was chosen as the working pipeline of the ML model, because it contains over 40 regression and classification models. A two-step prediction approach was adopted, integrating base and meta-learners (Fig. 3A). To prepare the data, 80% of the dataset was allocated for training, while the remaining 20% was kept aside for final testing.

In the first step of the process, the regression models were trained to predict microfluidic resistance on the basis of selected features. Specifically, 80% of the training portion (equivalent to 64% of the entire dataset) was used for model training, and the remaining 20% (16% of the entire dataset) served as an internal test set. All regression models were trained with their default settings, and the discrepancy between the predicted and actual resistance values was used to rank them. Only those models with an adjusted *R*^2^ exceeding 0.8 and a test mean absolute percentage error (MAPE) below 0.3% proceeded to the next step as base learners (Table 1). To enable the second step, an extra column was added to the dataset that identified the “best” regressors for each maze design. Subsequently, multiple classification models were trained to determine which regression model would be optimal for a given set of features (detailed in section S4). After these classification models were tested, the one with the highest accuracy, bagging classifier, was chosen as a meta-learner.

Following this classification phase, the second step of the framework was initiated, wherein the best regressor for a given feature set was automatically selected, and microfluidic resistance was then estimated using that regressor. The results of this classification step are presented in Fig. 3A, where the percentage distribution indicates how much of the dataset was assigned to each regressor. This two-step ensemble was lastly evaluated on the untouched 20% of the dataset, which was never used during the training process, and the results are shown in Fig. 3B. The approach used all models with their default settings, avoiding any specialized hyperparameter optimization and thereby demonstrating its versatility across diverse datasets. The predicted resistances showed a test root mean square error (RMSE) of 0.19, representing a 40% improvement over the single best baseline model (XGBRegressor) as detailed in section S4. Figure 3C illustrates how the classification-driven assignment of regressors enabled the model to tailor predictions to specific input regions, improving overall model performance. Moreover, the framework operated in perfect alignment with the developed generative model, elevating its capacity to simplify the design of target resistances while maintaining 3D printability and structural robustness in the fabricated microfluidic channels.

### Generative model

**Fig. 4.**
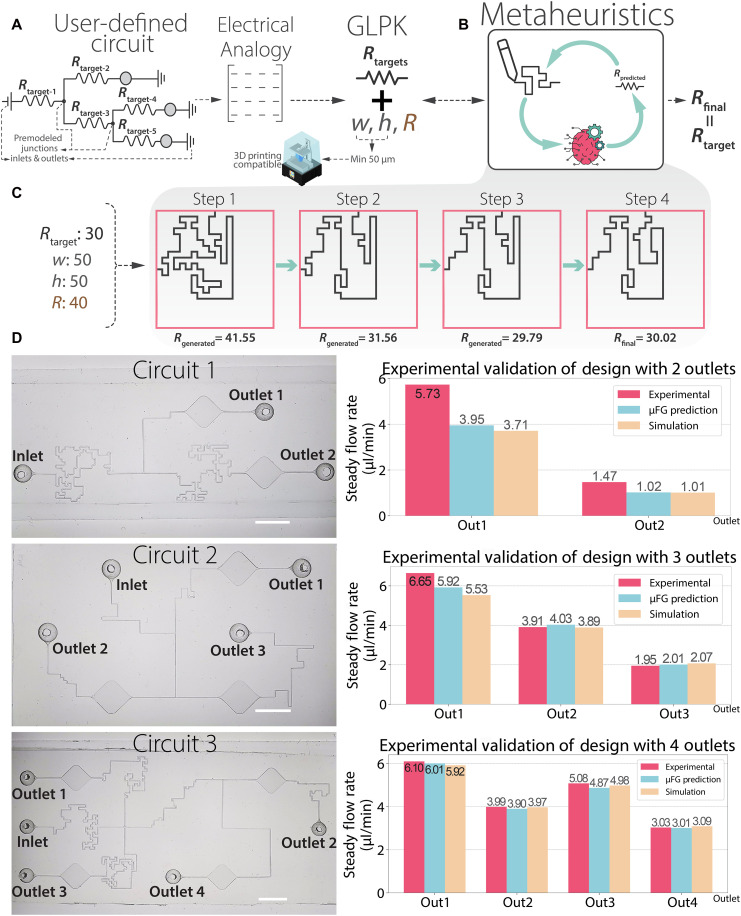
Circuit modeling workflow and experimental validation of μFG-generated microfluidic circuits. (**A**) Schematic of the circuit modeling algorithm. Based on user-specified flow rates, μFG constructs a matrix-based representation of the fluidic circuit using the electrical analogy. If the flow-rate inputs satisfy mass conservation at all nodes, then the required resistances for each channel are computed. An optimal solution is identified using the GNU Linear Programming Kit (GLPK) solver in combination with a metaheuristic search algorithm. (**B**) Overview of the metaheuristic design loop. The algorithm begins by randomly generating a maze structure with an initial parameter set. Then, ML-predicted resistance was converged to the target through step-by-step change of parameters. (**C**) Example of a generation process targeting a resistance of 30 mbar/(μl/min). The mathematical model constrains the circuit to a single cross-sectional geometry to avoid sudden internal transitions that may disrupt flow. The target resistance was reached with 99% accuracy by the fourth step. (**D**) Validation of μFG-generated circuit designs. Microscope images and outlet flow-rate measurements are shown for three circuits with two, three, and four outlets, respectively. Flow-rate comparisons (μFG prediction per experiment) revealed differences of 45.1 and 44.1% for circuit 1; 12.3, 3.0, and 3.0% for circuit 2; and 1.7, 2.3, 4.1, and 0.7% for circuit 3 ($R^{2}$ = 0.63, 0.95, and 0.99, respectively). The larger deviation in Circuit 1 arose from a pressure-threshold effect as explained in 2.5. Outlet-flow ratios closely matched their targets (3.90 versus 3.87 for circuit 1; 2.95:1.95:1.00 versus 2.96:2.02:1.00 for circuit 2; and 1.99:1.30:1.62:1.00 versus 2.00:1.33:1.67:1.00 for circuit 3), confirming accurate preservation of relative flow partitioning. Blue, yellow, and pink bars denote μFG-predicted, COMSOL-simulated, and experimental values, respectively. Scale bars, 5 mm.

Generation of microfluidic channels with specific resistances using an ML-powered instrument was targeted rather than only estimating channel resistance. A metaheuristic (tabu search) model was implemented alongside the instance-based ML model to achieve an iterative design generation process (Fig. 4B). The model included several constraints, the most critical being the realization of a target resistance. The *w*, *h*, and *L* predominantly determine resistance, while *r* and the number of corners have a subtler effect (fig. S5). Tabu search was used to randomly create mazes with specified *w*, *h*, and *r*. The instance-based ML model predicted the resistance of each generated maze.

The generative model’s further constraint was to produce designs adhering to practical limitations while meeting the desired resistance. Specifically, the minimum lateral resolution (40 μm) of the 3D printer imposed a lower bound on *w* and *h*, and reducing the number of corners helped avert manufacturing defects due to increased complexity. Moreover, unpredictable fabrication defects potentially increase a channel’s actual resistance, so the model was conditioned to approach the target resistance from below. If an initial estimate exceeded the target, then the discrepancy was multiplied by a factor, forcing a more aggressive correction in the next-generation step. These strategies helped account for potential manufacturing defects, which naturally produce higher measured resistances than theoretical estimates. Additionally, to expedite and refine the design process, a distract-and-repair method was introduced: If the predicted resistance fell within ±2 mbar/(μl/min) of the target, then the increments were changed by one-third, and if it lay within ±0.5 mbar/(μl/min), then it was altered 10-fold. This strategy enabled precise, incremental adjustments, consistently producing a final design in under 60 s on a typical consumer-grade laptop. Particularly, we tested on an Apple MacBook Air (CPU, Apple M2), and generations were complete in under 10 s. It is important to highlight that the duration of the generation is highly dependent on CPU power. The constraints of the generative model are further explained in the “Generative model” section.

Eight microfluidic channels spanning a resistance range from 0.5 to 45 mbar/(μl/min) were fabricated on the basis of geometries generated by μFG (section S6). These designs were created by specifying inlet pressures and target flow rates, and all flow values were selected to ensure operation within the laminar regime. The resistances shown in Fig. 3D, aimed at producing target values of 0.5, 1, 2, 4, 8, 10, 20, and 40 mbar/(μl/min), from left to right. Due to fabrication-induced dimensional deviations (Table 2), particularly in higher resistance channels, the experimentally measured resistances deviated from μFG’s targets by an average of ~28%. However, when actual printed dimensions were used as inputs into μFG’s ML model, the predicted resistances exhibited a much closer match differed from experimental values by only 12% on average, with a maximum deviation of 19% as presented in Fig. 3D. Comparisons with COMSOL simulations based on the same measured geometries showed similar agreement, with an average divergence of 13% and a maximum of 22%. These results confirm that μFG can reliably generate print-ready designs that achieve desired flow resistances when fabrication-aware feedback is incorporated. Detailed experimental and simulation protocols are provided in section S6.

**Table 2.** R^2^ RMSE, and MAPE values of the five best-performing regressors integrated into the proposed combined model from the training and testing procedures. These regressors (HistGradientBoosting, XGB, LGBM, KNeighbors, and GradientBoosting) were selected from 27 candidates (see table S3) for achieving adjusted *R*^2^ > 0.8, test MAPE < 0.3% with low computation time. This advanced performance not only highlights the predictive accuracy and computational efficiency but also demonstrates why a regressor-classification strategy is desirable, enabling the model to adaptively use the most suitable learner for each input condition and thereby maintain robust accuracy across the tested design space.

Regressor	Adjusted *R*^2^	*R* ^2^	Train RMSE	Test RMSE	Train MAPE (%)	Test MAPE (%)	Time (s)
HistGradientBoosting	1	1	0.34	0.34	0.02	0.02	0.94
XGB	1	1	0.31	0.31	0.02	0.02	0.61
LGBM	1	1	0.34	0.34	0.02	0.02	0.42
KNeighbors	1	1	0.39	0.57	0.01	0.02	0.65
GradientBoosting	1	1	0.52	0.52	0.05	0.05	14.79

### μFG interface and mathematical modeling of circuits

An .exe software was developed to let users specify a microfluidic pattern, target flow rates, and an inlet pressure (Fig. 1A). Flow rates, rather than resistances, were chosen to enhance usability. First, the software checks if the user-provided values uphold conservation of mass, which governs the constraints of the microfluidic circuit. It then constructs a circuit matrix using an electrical analogy and computes channel resistances that produce the desired flow distribution at the specified inlet pressure. The main purpose is to assign each branch’s resistance so that the user-defined flow rates are satisfied under the given pressure conditions.

Because there are more unknowns than equations, the resulting matrix system is nonsquare, introducing free variables and multiple potential solutions. This arises from uncertain intermediate pressures in branching sections. To address these uncertainties and arrive at a unique, practical solution, constraints and objectives were defined. The combined system, circuit matrix plus constraints, is then submitted to the GNU Linear Programming Kit (GLPK) solver to find an optimal set of resistances (Fig. 4A).

Practicality was a key consideration during design generation. The microfluidic circuit must maintain uniform *w*, *h*, and *r* to ensure a more robust structure, because abrupt dimensional changes can trigger undesired flow disturbances and unintentionally elevate overall resistance. Hence, *w*, *h*, and *r* were fixed at the outset to match the printer’s capabilities. The minimum lateral and vertical resolutions of 50 μm dictated that channel widths remain above the printer’s threshold. Additionally, limiting abrupt corners helped avoid manufacturing defects, given that straighter channels typically print more reliably.

Mixed integer linear programming (MILP) was used to incorporate such discrete choices into the optimization. The solution space was restricted to integer combinations of key parameters—in this case, channel *w*, *h*, and *r*—so each feasible combination has characteristic lower and upper resistance bounds within a given unit cell area. The lower bound corresponds to the shortest path, whereas the upper bound reflects the longest, most intricate path. Four reference mazes (two from a west inlet to an east outlet and two from west to north) were defined to represent these extremes. In essence, MILP transformed the problem’s solution space into discrete integer values, making it particularly well suited for scenarios that require specific, integer-based decisions; here, the selection of *w*, *h*, and *r* combinations. This process ensured that each possible combination is systematically evaluated, yielding an optimal design that meets user requirements and remains practical for manufacturing.

The overarching goal was to reduce the complexity of the resulting microfluidic network while still fulfilling the specified flow requirements. To accomplish this, the objective function minimized *F*, which is defined as the difference between the circuit’s highest resistance and the lower bound for that selected combination of *w*, *h*, and *r*. By compelling the most demanding channel to be as close as possible to its simplest, shortest-path form, the overall complexity of the circuit remains low while user-defined flow and pressure conditions are satisfied. Equations and additional details of this mathematical model are provided in the “Mathematical model” section.

Once the solver determines the needed resistances, the μFG interface deploys the ML-assisted metaheuristic generative algorithm to implement each resistance in practice (Fig. 4, B and C). Then, the final circuit is presented to the user along with generated final flow rates through each section of the circuit. The .exe software can be downloaded through our lab repository and used at will.

### Experimental validation of ML-generated microfluidic circuits

The software exports 3D printable .stl files of the designed microfluidic channels once the mathematical model determined the placement of specific resistances and their interconnections. To enable these channels and their junctions in the final 3D model, T- and cross-junctions, inlets, and reservoirs were predesigned in CAD software and then integrated into the application (detailed in section S5). The minor resistances introduced by these junction elements were evaluated using COMSOL simulations and factored into the circuit matrix. Two types of 3D model outputs are offered: a standalone microfluidic circuit, suitable for direct 3D printing, and a mold version (by selecting the “Add Base” feature), which is ideal for 3D printed soft lithography. In our experiments, we used the mold-based output to facilitate downstream fabrication steps.

Three different microfluidic circuits with single inlets delivering fluid to either two, three, or four channels were generated using the software and tested. Flow-rate sensors were attached at the inlet and relevant outlets (details in section S7). Each chip was carefully filled with water (bubble free) and pressurized until a steady flow regime was reached, after which flow rates were recorded for 300 s. Results of these experiments are summarized in Fig. 4D.

The first circuit, featuring two outlets, was intended to split an inlet flow of 4.97 μl/min into 3.95 and 1.02 μl/min at 10 mbar. Measured rates were 5.73 and 1.47 μl/min, yielding a 3.9 ratio that closely matched the targeted distribution share. However, if the flow rate in one outlet fell below 1.4 μl/min in the channel with higher resistance, then flow occasionally ceased altogether in that channel, likely due to the surface roughness of the 3D printed channels. A slight increase in inlet pressure mitigated this effect, but smoother channel surfaces are expected to eliminate it entirely. The second circuit, containing three outlets, aimed to distribute 12.7 μl/min at 40 mbar into flows of 5.92, 4.03, and 2.01 μl/min. Measurements of 6.65, 3.91, and 1.95 μl/min demonstrated that the ML-generated design achieved over 90% accuracy. The third design, with four outlets, was targeted to apportion 18.2 μl/min at 60 mbar into flows of 6.10, 3.99, 5.08, and 3.03 μl/min. Steady-state values of 6.0, 3.91, 4.87, and 3.01 μl/min confirmed 95% accuracy.

Because flow-rate data can be affected by sensor variability, COMSOL simulations of the same μFG-generated circuits were also performed (fig. S10). These agreed with experiments within 92% accuracy (Fig. 4D), reinforcing the reliability of the autonomously designed circuits. Overall, the results illustrate that μFG can autonomously produce circuits tailored to user-defined flow distributions. The ML-supported generative algorithm successfully creates target resistances, and its integration with an electrical circuit analogy facilitates automated design. Even the most complex circuit tested, one featuring three outlets, required only about 3 min to generate on a standard computer, underscoring both the efficiency and practical applicability of this approach.

After confirming that μFG-generated circuits achieved the target flow distributions, we applied the framework to physiologically relevant systems as a proof of concept. Traditionally, complex robotic or pneumatic systems are required to replicate organ-level pharmacokinetics and interorgan fluid communication on microchips (*59*–*61*). Edington *et al*. (*62*) reported pneumatically controlled “4-way,” “7-way,” and “10-way” interconnected microphysiological systems (MPSs) that emulate tissue-specific flow partitioning. To demonstrate μFG’s scalability and applicability to such contexts, these flow-partitioning ratios were entered into μFG, and corresponding circuit designs were generated (section S8). The resulting geometries closely matched the intended flow distributions, showing strong correlations with target values ($R^{2}=0.991$ for 4-way, $R^{2}=0.999$ for 7-way, and $R^{2}=0.999$ for 10-way MPSs). These analyses highlight μFG’s ability to autonomously design massively parallel microfluidic circuits that reproduce complex physiological flow architectures without manual tuning of excessive pneumatic pumps. The platform thus offers a practical and accessible route for researchers across disciplines to model flow partitioning and generate manufacturable microfluidic designs for biological applications.

## DISCUSSION

We introduced μFG, a microfluidic design tool that combines ML with algorithmic optimization to automatically generate channel layouts matching user-defined flow rate and distribution requirements. By eliminating the need for iterative, manual tuning, μFG makes precision microfluidic design accessible to nonexpert users while maintaining the flexibility and performance typically associated with expert-crafted systems.

This computational framework enables the generation of chip designs that replicate physiologically relevant flow rate–dependent conditions, such as controlled perfusion and shear stress in tissue models (*63*), metabolite monitoring in cancer-on-chip systems (*64*), interorgan cross-talk studies between microtissues and organoids (*65*), and quantitative tracking of extracellular vesicle transport in gut-brain-axis-on-chip platforms (*66*). Additionally, μFG supports proportional flow distribution across circuit branches, a critical feature for mimicking physiological blood supply to distinct tissues (*67*). Previously, the need to reproduce complex physiological flow conditions on-chip was often addressed using elaborate robotic fluid–handling systems or extensive external tubing. In contrast, the MPS circuits generated by μFG achieved user-defined flow rates and distributions solely through passive pressure control and channel geometry, without requiring any auxiliary mechanical components.

Prior efforts toward design automation have laid valuable groundwork but remained limited in scope or generalizability. For instance, the uFlow tool by Stoecklein *et al*. (*68*) enabled nonexpert users to visualize micropillar array effects through custom simulations. Similarly, the Grover lab developed a performance-driven database tool for predefined microfluidic grid configurations (*69*). Users could input a target concentration distribution for two-inlet, three-outlet chips, and the system would return the closest-performing design from its dataset. However, these tools were constrained by fixed layouts and did not include structure generation or embedded performance prediction. Likewise, while the Densmore group’s 3DuF platform enabled users to rapidly create layouts including valves, chambers, and other components, yet it did not offer integrated tools to evaluate device performance during the design process (*70*). These limitations hindered broad adoption for use cases requiring performance-driven customization.

ML models have been applied to predict various performance parameters in microfluidic systems, such as estimating droplet sizes (*71*–*73*) and predicting the dimensions of microfluidics-generated nanoparticles (*74*). In a complementary direction, design optimization has been explored through algorithmic approaches, including the use of Bayesian optimization for enhancing micromixing efficiency (*75*) and genetic algorithms for improving capillary network architectures (*76*). While these approaches advance either performance prediction or design refinement, they fall short of enabling fully automated generation of microfluidic structures that meet user-defined performance criteria.

Lashkaripour *et al*. (*51*) introduced design automation of fluid dynamics (DAFD), a tool that leverages neural network-based prediction combined with iterative algorithms to automatically generate droplet generator design parameters, such as geometry and flow conditions, that meet user-defined droplet sizes and production rates. In a subsequent study, they enhanced the predictive performance of DAFD by combining neural networks and boosted decision trees, thereby improving the accuracy and robustness of the model’s output (*52*). However, the system remained limited in terms of the integration of search algorithms for advanced automation of the design process and also did not provide users with ready-to-use models.

In terms of computational efficiency and predictive accuracy, μFG outperforms prior ML-based design tools. DAFD achieves MAPEs of ~7 to 23% for droplet diameter and generation rate, with an enhanced hybrid model, combining neural networks and boosted decision trees, improving this range to 5 to 17% on test data. For automated design, DAFD delivers user-specified droplet performance within 4 to 8% MAPE for diameter and 12 to 38% for generation rate, although this process typically requires up to 5000 iterative evaluations to converge on a single solution. The 3DuF platform, while capable of interactive layout construction with complex devices in under 20 min of user time, lacks integrated performance prediction and depends on external simulation for validation. In contrast, μFG achieves subpercent error in hydraulic-resistance prediction (MAPE of 0.009% and RMSE of 0.19) and generates fully functional designs within seconds, eliminating the need for manual iteration or external evaluation. Together, these features position μFG as a practical, highly accurate alternative to existing ML-based design frameworks.

Beyond its predictive performance, μFG integrates a two-step ML resistance-prediction model with metaheuristic generative modeling to achieve end-to-end automated design. By coupling this predictive engine with metaheuristic optimization, μFG achieved target microfluidic resistance generation with 89% accuracy. The framework then uses electrical circuit analogies to convert user-defined network patterns into solvable matrices. The current implementation supports networks with one inlet and multiple outlets under atmospheric pressure, but the framework is extensible to include multiple inlets, intermediate pressure constraints, or boundary pressures differing from atmospheric values. The underlying mathematical model itself is liquid-agnostic and can, in principle, operate with any Newtonian fluid. In this study, however, the ML algorithms were trained on datasets generated using water, making the present implementation quantitatively optimized for water. Because the governing equations remain unchanged, μFG can be readily adapted to other liquids simply by regenerating the training dataset under their respective physical conditions (e.g., viscosity and density). This adaptability will help the framework maintain predictive accuracy across different fluids without modification of the core mathematical model.

Following circuit computation, μFG automatically outputs a downloadable 3D model in the standard .stl format. The universal compatibility of .stl files enables users to visualize and modify the generated geometries using any commercial or open-source 3D modeling software, eliminating the need for SolidWorks or other proprietary tools, and facilitating direct integration into diverse fabrication pipelines. While we used 3D printing for our experimental validations, the resulting designs are readily adaptable to micromilling or soft lithography with minimal modifications. Fabrication constraints, such as printer resolution, were incorporated into the design process to ensure manufacturability. The mathematical solver systematically favors lower resistance values across the entire circuit layout, because reduced hydraulic resistance is typically achieved through wider channel cross sections and less geometrically complex pathways. This preference leads to shorter, straighter, and broader channels, which are inherently more robust to fabrication variability and easier to manufacture with high fidelity. In parallel, the metaheuristic algorithm approaches each target resistance conservatively from below, a strategy that compensates for the tendency of fabrication errors to increase actual resistance. Together, these two features, simplified geometry and conservative resistance estimation, enabled μFG to achieve >90% experimental performance accuracy in 3D printed circuit validation tests. In future iterations, the software could be enhanced by incorporating noise-introduced training data, following a “model vaccination” strategy in which exposure to slightly perturbed data helps models generalize to real-world imperfections (*77*). Averaging predictions across noisy and clean values during training could further improve μFG’s fabrication tolerance and experimental reliability.

Furthermore, a more general design-rule-checking framework could further expand μFG’s adaptability by parametrizing fabrication-specific defect profiles for different manufacturing methods. Such a system could allow users to define characteristic tolerances—such as layer misalignment, surface roughness, or local dimensional deviations—associated with their chosen fabrication process. The correction algorithm could then adjust accordingly, compensating for technique-specific sources of error. While these extensions would primarily refine the extent of geometric correction rather than alter the core resistance-preserving logic, they would provide a unified interface for tailoring μFG to a broader range of fabrication technologies, including photolithography and computer numerical control (CNC) micromilling. Nonetheless, the aggressive correction strategy implemented in μFG has proven effective and can be readily generalized to fabrication processes where defect formations mainly increase the resistance.

Critically, μFG is open-source and modular, encouraging users to explore the integration of additional microfluidic components, such as micromixers, gradient generators, or flow-focusing junctions. While these capabilities are not yet implemented within the automated workflow, the framework can be extended for further automation. Future studies might expand μFG’s applicability to multi-inlet and feedback architectures or to devices involving coupled flow–diffusion behavior such as mixers and gradient generators. These extensions would primarily require unique datasets, along with corresponding refinements of the mathematical and optimization models to handle coupled pressure and concentration constraints. Such developments would enable μFG to design more complex networks while maintaining its data-driven, performance-aware automation.

By abstracting core design complexities, μFG lowers the barrier for entry and promotes broader engagement from researchers across disciplines, particularly those in diagnostics, biotechnology, and education. In summary, μFG combines ML-enhanced prediction, metaheuristic modeling, and fabrication-aware optimization into a unified platform that supports accessible, accurate, and fully automated microfluidic design. This paradigm shift in design methodology has the potential to democratize microfluidics and accelerate innovation across diverse application areas.

## MATERIALS AND METHODS

### Microfluidic maze definition

A matrix-based representation was used to construct the maze using a custom Python script. The matrix encoded the coordinates that the algorithm followed to generate the path. The coordinate system was defined over a 10 mm–by–10 mm area with a spatial resolution of 0.5 mm, resulting in a 20 by 20 grid (Fig. 2A). For example, the inlet was fixed at coordinate (0, 5), while the outlet was positioned at either (10, 5) or (5, 10). At each control step, the direction to proceed was randomly selected on the basis of the output of a “Shortest Path” algorithm, ensuring a unique solvable path and rapid generation. The algorithm avoided self-intersections and boundary crossings during generation. Once a feasible path connecting the inlet and outlet was generated, the matrix was converted into a coordinate-based path representation.

### Simulations and experimental validation of simulations

The Laminar Flow Module of COMSOL Multiphysics (v. 6.0, COMSOL Inc.) was used to create a dataset containing 328,320 data points. The simulation method was verified experimentally by manufacturing microfluidic channels with 3D printed soft lithography. Pr110-385 by CadWorks3D (Canada), a digital light processing–based resin printer, was used to print molds from the company’s master mold resin. Sylgard 184 Silicone elastomer kit (Dow Corning, USA) was used to form polydimethylsiloxane channels from these molds. The details of the simulation and manufacturing are thoroughly explained in sections S1 and S2. Eight different microfluidic mazes with geometrical combinations described in Fig. 2B were used to validate our simulation method.

### Data generation

Coordinate-based maze pathways were imported into COMSOL Multiphysics (v. 6.0, COMSOL Inc.) for CAD modeling and geometry generation. Each microfluidic maze was defined by a rectangular cross section characterized by width (*w*) and height (*h*) (Fig. 2A). To ensure smooth transitions and avoid meshing artifacts, a fillet radius (*r*) was applied to all sharp corners. Parameters influencing hydraulic resistance included the total channel length (*L*; computed from the coordinate path), number of corners, and the values of *w*, *h*, and *r*.

Mazes were randomly generated using unique combinations of these parameters while conforming to dimensional constraints. Redundant and mirrored designs were excluded, yielding a final library of 1710 unique geometries. The specific values of *w*, *h*, and *r*, along with the observed ranges for *L* and number of corners, are provided in Table 1.

This library was imported into MATLAB (MathWorks Inc., R2023b), and a bidirectional link with COMSOL was established via the LiveLink interface. A parametric simulation workflow was executed for each maze geometry across 192 combinations of *w*, *h*, and *r*, resulting in 328,320 unique simulations. For each instance, flow rate and hydraulic resistance values were computed and stored alongside the corresponding geometric parameters. This dataset served as the foundation for training and evaluating the ML models developed in this study.

### Resistance prediction

To estimate the hydraulic resistance of microfluidic maze designs based on geometric features, we developed an ML pipeline using Python. Each entry in the dataset included five input features: *w*, *h*, *r*, *L*, and the number of corners. Simulated resistance values were used as the target variable. The dataset was randomly divided into training (80%) and test (20%) subsets using stratified sampling. The training portion was further split into training (80%) and validation (20%) sets to support model selection. All model development was carried out using the open-source LazyPredict library, which provides a standardized interface for evaluating a broad set of regression and classification algorithms from scikit-learn.

In the first stage of the pipeline, 27 regression models were trained with default hyperparameters to predict resistance from geometric inputs (table S3). Model performance was evaluated on the basis of absolute prediction error. For each training instance, the regression model that produced the lowest error was recorded as the optimal base learner.

To enable model-specific inference, a second-stage classification task was introduced. The best-performing regressor for each sample was used as the label to train a set of classification models tasked with predicting the most appropriate base learner from the input features. A total of 20 classification models were tested in this stage, and the one achieving the highest accuracy, bagging classifier, was selected as the final meta-learner (section S4).

All data processing and statistical operations were performed using the NumPy and Pandas libraries. Source code and implementation instructions for reproducing the full pipeline are available at https://github.com/dxbiotech/Microfluidics-Resistance-ML.

### Generative model

A metaheuristic algorithm based on tabu search was implemented and integrated with a ML-based resistance prediction model. The generative framework, developed in Python, aims to design mazes with target resistance values while satisfying geometric constraints such as width, height, and fillet radius. To achieve this, it uses a metaheuristic optimization process based on sabu search, where two distinct neighbor functions, N1 and N2, iteratively modify the current maze configuration. Both functions operate on the principle of destructing and then repair sections of the maze path to explore design alternatives. N1 randomly selects two indices within the path and deletes the segment between them, subsequently repairing it using one of three strategies: shortest, longest, or random. This function is generally used for local modifications that fine-tune the internal structure of the maze. In contrast, N2 targets a broader restructuring by focusing on the endpoints of the path, either the beginning or the exit location, and deletes the path segment connecting a randomly selected point to one of these endpoints. It then repairs the gap using a similar set of strategies. Both N1 and N2 rely on weighted random selections that prioritize structurally meaningful areas of the maze, such as corners or central segments. Each freshly generated maze is evaluated by a fitness function, which measures the deviation of its predicted resistance, calculated via the ML model, from the desired value, with additional penalization applied if the resistance is too high. This process allows the algorithm to gradually converge on an optimal or near-optimal maze design that satisfies the resistance and geometric constraints. The algorithm explores 50 neighbor designs in each step and avoids repeating recent solutions by using a tabu list of size 10. The procedure was repeated until the target resistance reached 99% absolute proximity (Fig. 4C). Notably, each generation loop was limited to a maximum of 120 s and 50 generations. The process was ended after reaching target resistance and returned the final channel design.

### Mathematical model

A mixed-integer linear programming (MILP) model grounded in an electrical circuit analogy was formulated to algorithmically generate microfluidic networks with prescribed resistance values. Pressure points were defined as inlets, outlets, and division junctions within the network, and lines were modeled as segments connecting consecutive pressure points. Each line was composed of resistance unit cells categorized as either straight or corner unit cells, depending on their geometric orientation. The overall circuit geometry was defined by a global combination of design parameters—*w*, *h*, and *r*—which were uniformly applied across all segments.

The objective (*F*) of the MILP model was to minimize the complexity of the resulting circuit by encouraging the selection of low-resistance configurations. Specifically, the model minimized the maximum deviation of assigned resistance values from their respective lower bounds. This approach biases the solution toward designs that are closer to the lower resistance limit of each geometric configuration, which, in turn, simplifies downstream fabrication and improves 3D printability.

The model involved three sets:

1. I: Set of indices for lines. ($I=\left\{1,2,3,\right\}$)

2. $I^{\prime }$: Set of indices for the lines with exit pressure points from the circuit at the end. ($I^{\prime }\subseteq I$)

3. J: Set of indices for combinations.

In addition, parameters defined over these sets included:

1. $P_{0}$: Entry pressure value.

2. $Q_{i}$: Flow rate on the line i. (i∈I)

3. $n_{i}^{s}$: Number of straight unit cells in the line i. (i∈I)

4. $n_{i}^{c}$: Number of corner unit cells in the line i. (i∈I)

5. $l_{j}^{s}$: Lower bound of resistance on the straight unit cell has combination j. (j∈J)

6. $u_{j}^{s}$: Upper bound of a resistance on the straight unit cell has combination j. (j∈J)

7. $l_{j}^{c}$: Lower bound of a resistance on the corner unit cell has combination j. (j∈J)

8. $u_{j}^{c}$: Upper bound of a resistance on the corner unit cell has combination j. (j∈J)

9. $d_{j}$: Resistance value of a pressure point has combination j. (j∈J)

The model was subject to this set of equations

$$\sum_{j\in J}x_{j}=1$$ (1)

$$\begin{array}{cc} P_{0,j}=P_{0}\cdot x_{j} & \forall j\in J \end{array}$$ (2)

$$\begin{array}{cc} P_{i,j}=0 & \forall i\in I^{\prime },\forall j\in J \end{array}$$ (3)

$$\begin{array}{cc} Q_{i}\cdot \left(R_{i,j}+2\cdot d_{j}\cdot x_{j}\right)=P_{i-1,j}-P_{i,j} & \forall i\in I,\forall j\in J \end{array}$$ (4)

$$\begin{array}{cc} n_{i}^{s}\cdot R_{i,j}^{s}+n_{i}^{c}\cdot R_{i,j}^{c}=R_{i,j} & \forall i\in I,\forall j\in J \end{array}$$ (5)

$$\begin{array}{cc} R_{i,j}^{s}-l_{j}^{s}\cdot x_{j}\leq F_{j} & \forall i\in I,\forall j\in J \end{array}$$ (6)

$$\begin{array}{cc} R_{i,j}^{c}-l_{j}^{c}\cdot x_{j}\leq F_{j} & \forall i\in I,\forall j\in J \end{array}$$ (7)

$$\begin{array}{cc} F_{j}\leq F & \forall j\in J \end{array}$$ (8)

$$\begin{array}{cc} l_{j}^{s}\cdot x_{j}\leq R_{i,j}^{s}\leq u_{j}^{s}\cdot x_{j} & \forall i\in I,\forall j\in J \end{array}$$ (9)

$$\begin{array}{cc} l_{j}^{c}\cdot x_{j}\leq R_{i,j}^{c}\leq u_{j}^{c}\cdot x_{j} & \forall i\in I,\forall j\in J \end{array}$$ (10)

$$\begin{array}{cc} R_{i,j}\geq 0 & \forall i\in I,\forall j\in J \end{array}$$ (11)

$$\begin{array}{cc} P_{i,j}\geq 0 & \forall i\in I,\forall j\in J \end{array}$$ (12)

$$\begin{array}{cc} F_{j}\geq 0 & \forall j\in J \end{array}$$ (13)

F≥0 (14)

$$\begin{array}{cc} x_{j}\in \left\{0,1\right\} & \forall j\in J \end{array}$$ (15)

Here, the decision variables are explained as follows:

1. $P_{i,j}$: Pressure value at the end of the line i has combination j. (i∈I, j∈J)

2. $x_{j}$: 1, if the combination j is selected for the circuit; 0, otherwise. (j∈J)

3. $R_{i,j}^{s}$: Resistance value of a straight unit cell in line i has combination j. (i∈I, j∈J)

4. $R_{i,j}^{c}$: Resistance value of a corner unit cell in line i has combination j. (i∈I, j∈J)

5. $R_{i,j}$: Total resistance value in line i has combination j. (i∈I, j∈J)

6. $F_{j}$: The most significant difference between a resistance value in the circuit with combination j and the lower bound of the same combination j. (jJ)

7. F: The maximum of $F_{j}$’s.

The constraint in Eq. 1 ensures that only one combination from the set of available design configurations can be selected for the entire circuit. Equation 2 enforces that the entry pressure for the selected combination equals the predefined inlet pressure $P_{0}$, while Eq. 3 ensures that all outlet pressure values are fixed at 0 mbar, consistent with typical microfluidic boundary conditions. Equation 4 captures the pressure-flow relationship based on Ohm’s law analogy (Q·R=∆P), where the term $2\cdot d_{j}\cdot x_{j}$ accounts for additional resistance contributions from divisions, inlets, or outlets located at the beginning and end of each line. The total resistance of a line, defined as the sum of resistances from corner and straight unit cells, is enforced by Eq. 5. Equations 6 to 8 collectively compute the maximum deviation from the lower resistance bound across all combinations, thereby enabling the model to minimize design complexity. Last, Eqs. 9 to 15 define the feasible bounds for all decision variables, including pressure, flow rate, resistance, and binary selection indicators.

The MILP model was implemented in Python and solved using GLPK via its Python application programming interface (API). A typical instance included approximately *N* binary variables and *M* continuous variables, where *N* and *M* scaled with the number of circuit lines and the cardinality of the combination set. The model output defined both the segment-wise resistance configuration and the global resistance distribution for the entire maze. These results were later translated into fabrication-ready CAD geometries that conformed to the required hydraulic behavior while remaining within the bounds of 3D printability.

### μFG software

The standalone executable software was developed using the tkinter library and features an intuitive graphical user interface for constructing custom microfluidic circuits. Users can design layouts by selecting and placing predefined components, including channel segments, reservoirs, T- and cross-junctions, inlets, and outlets. The predefined components were designed in SolidWorks (Dassault Systèmes, France) and loaded into the software as .stl models (detailed in section S5). To place an element, the user must first activate it by clicking the blue “Select” button corresponding to the desired component. Channel segments, including rotated variants, can be oriented using the “Rotate” button. Once activated, elements are placed on the design grid via left-click. To remove an existing component, the user can middle click directly on the unit cell where the element was placed.

It is essential that junctions are connected to other features through at least one channel segment (straight or corner) to ensure proper resistance assignment by the underlying algorithm. If an inlet or outlet is connected directly to a junction without an intermediate segment, then the software will not be able to compute the corresponding resistance, resulting in an invalid circuit configuration.

Once the layout is defined and target flow rates are assigned, the user initiates circuit synthesis by clicking the “Build” button. The software then performs computational modeling to generate a circuit design that satisfies the specified flow constraints. After a short calculation period, the resulting circuit is displayed alongside the predicted flow rates for each outlet, including a comparison to the user-defined targets. If the solution is satisfactory, the user can export the design by clicking the “Download” button, which generates a 3D printable .stl file of the circuit. Additionally, the “Add Base” button enables export of the circuit embedded in a mold base, suitable for soft lithography or mold-based replication.

### Experimental evaluation of the μFG-generated microfluidic circuits

Experimental validation of μFG-generated microfluidic circuits was performed using custom flow setups tailored for multi-outlet designs. Flow-rate measurements were obtained using high-sensitivity microfluidic flow sensors connected to an OB1 pressure controller, enabling simultaneous monitoring of inlet and outlet streams. Circuit configurations with two, three, and four outlets were tested across a range of input pressures. In each case, recorded flow distributions were compared to target values generated by the μFG model. The system was allowed to reach steady-state before data acquisition, and flow-rate measurements were averaged over 5-min intervals. The resulting mean flow rates are presented in the plots of Fig. 4. Detailed experimental procedures, sensor arrangements, and flow profiles are provided in section S6.

## Supplementary Materials

### This PDF file includes:

Supplementary Informations S1 to S8 Figs. S1 to S15 Tables S1 to S6
