# Supplementary material for: ML-automated microfluidic circuit design
Source: Sci Adv. 2026 Jan 28;12(5):eaea7598. doi: 10.1126/sciadv.aea7598 (PMC12851026; doi:10.1126/sciadv.aea7598)
Supplement: Supplementary file 1 — Supplementary Informations S1 to S8 Figs. S1 to S15 Tables S1 to S6 [file sciadv.aea7598_sm.pdf]

Science Advances
AAAS

Supplementary Materials for

ML-automated microfluidic circuit design

Mehmet Tugrul Birtek *et al.*

Corresponding author: Savas Tasoglu, stasoglu@ku.edu.tr

Sci. Adv. **12**, eaea7598 (2026)
DOI: 10.1126/sciadv.aea7598

This PDF file includes:

Supplementary Informations S1 to S8
Figs. S1 to S15
Tables S1 to S6

Supplementary Information

ML Automated Microfluidic Circuit Design

Mehmet Tugrul Birtek, Vural Aktas , Bora Aktas, Ahmed Choukri Abdullah, Aydogan Ozcan and Savas Tasoglu

SI.1. Simulations

COMSOL Multiphysics was utilized to optimize the simulations of the microfluidic channels. Coordinates of randomly generated mazes were imported into COMSOL to model the complex geometries accurately by transferring polygon coordinates from Python. Fillets were then created at the corners of the polygon to smooth sharp edges. A thickness corresponding to the channel width was assigned to the polygon to establish the channel geometry. Two edges were selected to define the inlet and outlet regions of the flow.

Water was assigned as the domain material, while polydimethylsiloxane (PDMS) was used for the walls, reflecting the materials used in the experimental setup. The Laminar Flow physics interface was employed, utilizing the incompressible Navier-Stokes equations with inertial terms neglected to model Stokes flow conditions accurately. A shallow channel approximation was applied by specifying the channel thickness as the height, simplifying the three-dimensional flow to a two-dimensional problem. The no-slip boundary condition was imposed on all walls, and pressure boundary conditions at the inlet and outlet were set.

User-controlled meshing settings were employed to maintain robust control over mesh quality. As illustrated in **SI Fig. 1b**, mapped meshing was applied to the main channel domain, while distribution meshing was used on boundary edges to accommodate complex geometric features. A mesh independence study, conducted on eight different microfluidic mazes spanning resistances of 8–68 mbar/(μL/min) (**SI Fig. 1a, c**), validated the simulation method for a broad range of flow resistances. The results showed that channel resistance values remained stable for element counts higher than 10. Based on these observations, an element count of 12 was chosen for subsequent simulations, ensuring both computational efficiency and accuracy in resistance calculations.

$$R = \frac{P_{in} - P_{out}}{Q} \qquad (s.1)$$

A stationary study was conducted to solve the flow equations under steady-state conditions. The results included calculations of the flow rate within the microfluidic channel and the hydraulic resistance, determined using formula **s.1.**, where P_{in} and P_{out} are the inlet and outlet pressures, respectively, and Q is the volumetric flow rate.

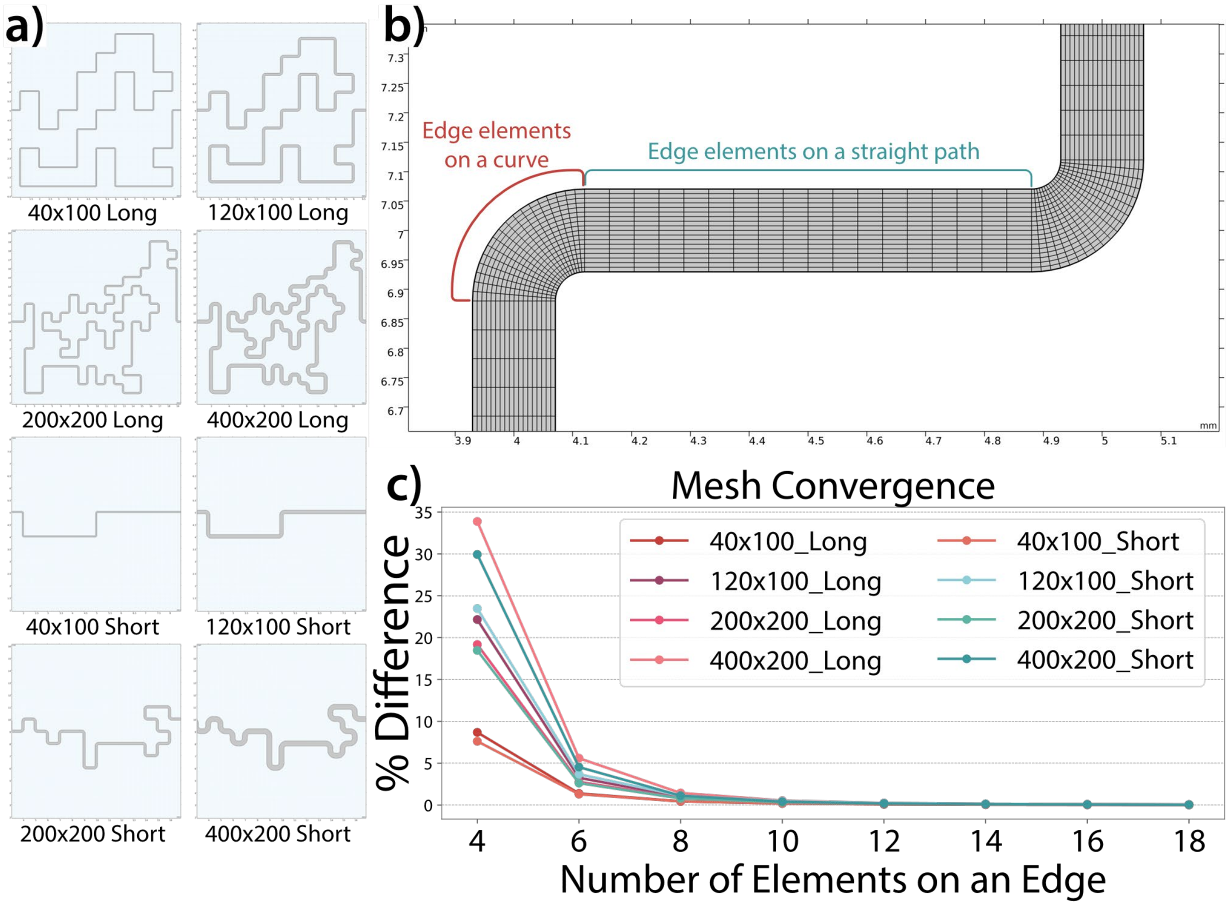

SI Figure 1. Convergence study of the meshing used in COMSOL simulations. (**a**) Eight different microfluidic mazes were created in COMSOL. The numeric labels in each design name represent the channel width and height (μm). Both long and short versions of each geometry were generated to span a broad range of flow resistance values. (**b**) Representative mesh structure on a 120 μm-wide channel segment, illustrating how surface elements (including curved and flat sections) are subdivided. The "number of elements" refers to the number of divisions along the edges of these surface elements. (**c**) Mesh convergence analysis. Each data point's percentage difference was obtained by comparing the simulation result at a given element count to that at the previous lower element count. For example, the point at four elements was compared to two elements. All models showed less than 0.1% difference for element counts above 10. Consequently, an element count of 12 was selected as the optimal balance between computational efficiency and simulation accuracy.

SI.2. Manufacturing Microfluidic Chips

The digital light processing (DLP)-based printer used for mold fabrication had a resolution specified by the manufacturer as 40 μm in the horizontal axis and 30 μm in the vertical axis. We selected the dimensions of the eight experimental validation mazes by considering the limitations of the 3D printer while aiming to include a range of resistances.

The coordinates of the randomly generated mazes were imported into computer-aided design (CAD) software, where a pool-shaped base was added to create molds suitable for casting. The molds were printed using the DLP printer, thoroughly washed with isopropyl alcohol (IPA) to remove any uncured resin and then cured in a UV chamber for 1 hour to ensure complete polymerization. Sylgard 184 polydimethylsiloxane (PDMS) was mixed with its curing agent at a ratio of 10:1 (w/w), poured into the molds, and degassed to remove any trapped air bubbles. The PDMS was then cross-linked by placing it in an 80 °C oven for 1 hour. After curing, the PDMS replicas were carefully removed from the molds, and inlets and outlets were punched through the PDMS. The punched PDMS chips were bonded to glass microscope slides via oxygen plasma activation. The process is graphically briefed in **SI Fig. 2**. The heights of the manufactured channels were measured using a Bruker DekTakXT surface profilometer

(Bruker, Germany) while the widths were measured with ImageJ analysis of microscope images.

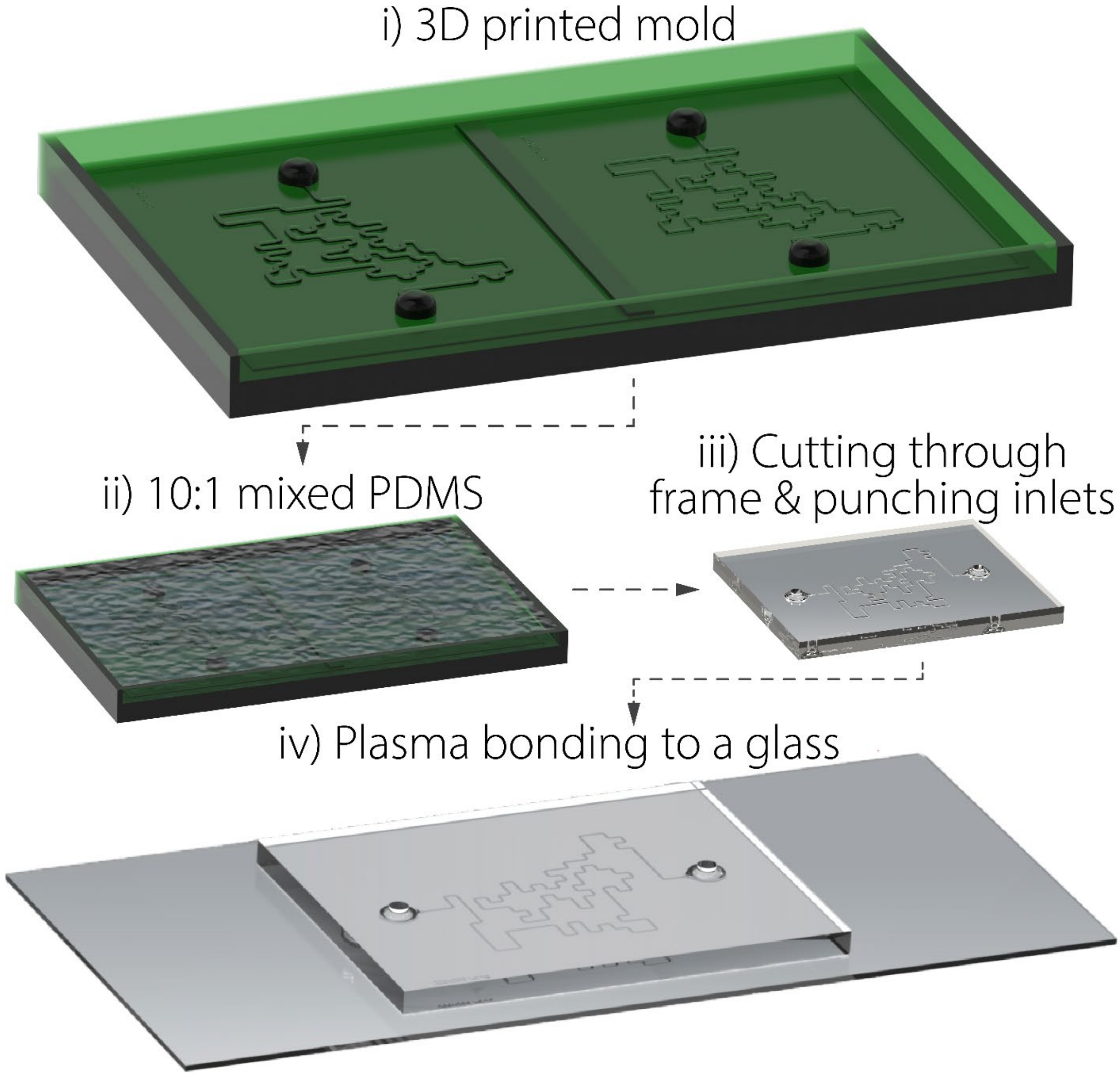

SI Figure 2. Demonstration of 3D printed soft lithography method for manufacturing microfluidic channels. (**i**) The 3D-printed mold features a pool shape to contain liquid PDMS, with incorporated microfluidic patterns defining channel geometry. (**ii**) PDMS is poured into the mold, degassed in a vacuum chamber, and crosslinked via heat treatment. The cured PDMS layer is then lifted from the mold. (**iii**) Sacrificial edges are trimmed to remove any deformed regions formed during lift-off. Inlet and outlet ports are subsequently punched to allow fluid flow. (**iv**) The finalized PDMS channel is plasma-bonded to a glass slide, completing the device assembly.

SI.3. Experimental Validation of Simulation Method

To validate the practicality of our simulation method, we developed an experimental setup designed to precisely measure the hydraulic resistance of microfluidic channels. Four randomly generated maze geometries were selected to represent a broad range of resistance values (**Fig. 2b**). As detailed in **SI Table 2**, each maze was fabricated with two distinct cross-sectional profiles, yielding a total of eight different designs. All experiments were conducted using an Elveflow OB1 pressure controller (Elvesys, France) and an MFS2 flow rate sensor.

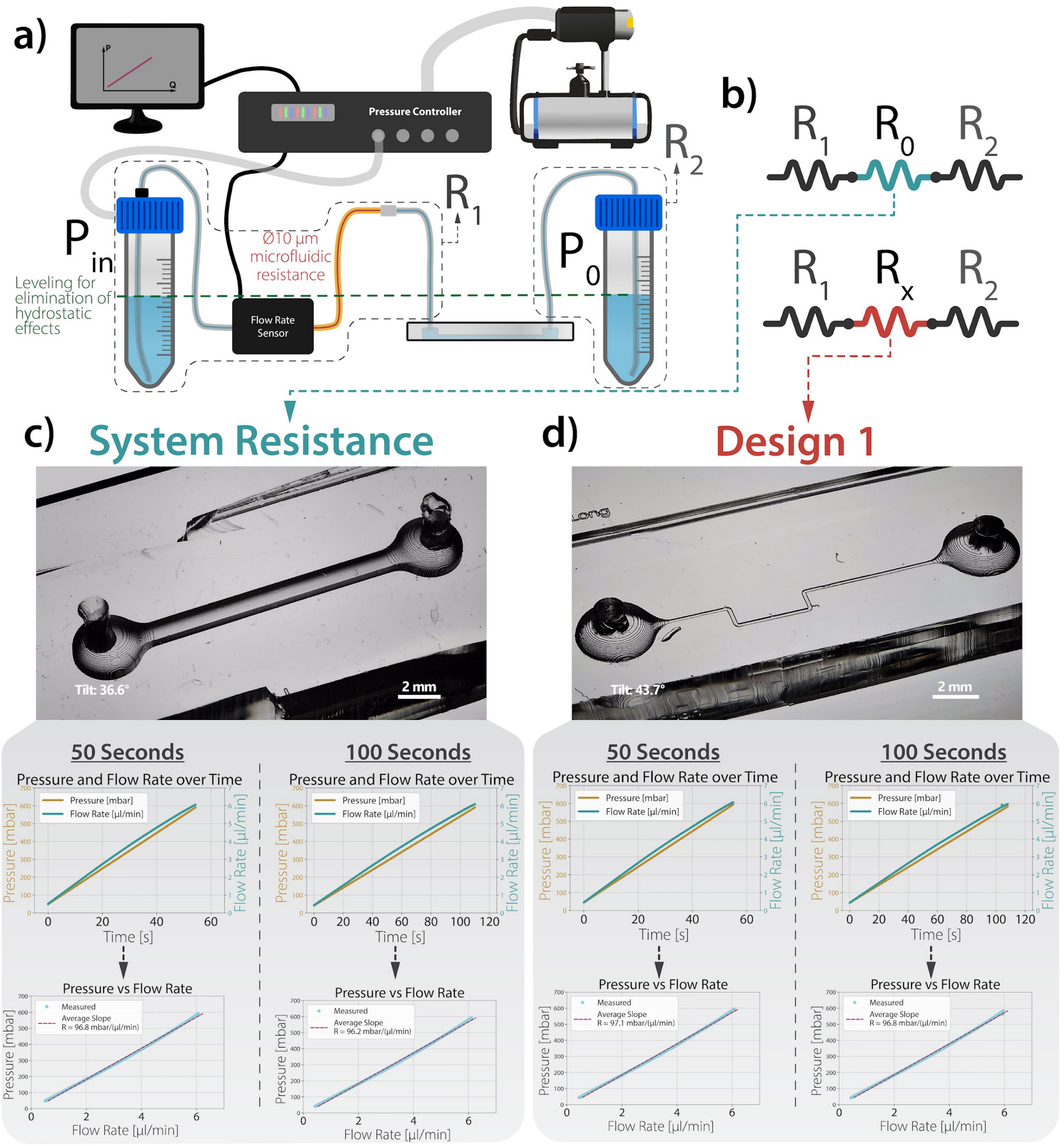

SI Figure 3. Experimental setup and methodology for measuring microfluidic channel resistances. (**a**) Illustration of the pressure-controlled experimental setup. A high-resistance tube was placed between the flow rate sensor and the tested microfluidic chip to buffer inlet pressure fluctuations. $\boldsymbol{R_1}$ and $\boldsymbol{R_2}$ denote the systematic resistances present in all experiments. (**b**) Electrical circuit analogy of the setup shown in (a). The tested microfluidic chip is connected in series with the high-resistance tubing, allowing total resistance to be modeled as a sum of individual components. (**c**) Reference system chip used for system resistance characterization. This low-resistance chip (800 × 800 µm cross-section, 10 mm length) was first tested to estimate the baseline system resistance. Two dynamic experiments were conducted: pressure was ramped from ~50 to ~600 mbar over either 50 or 100 seconds while recording both pressure and flow rate. Resistance was calculated from the slope of the resulting pressure–flow plots for each ramp duration. (**d**) Example measurement for Design 1 from **SI Table 1**. In addition to the two dynamic tests, steady-state flow rate at 400 mbar was recorded. The chip's resistance was computed as the average of three values—derived from 50-second ramp, 100-second ramp, and steady-state measurement. The system resistance (obtained in part c) was subtracted from this average to yield the final experimental resistance of the tested design.

To isolate the resistance of the microfluidic maze from the total system resistance, we modeled the fluidic path as a series of resistances (**SI Fig. 3b**). The fluidic system was designed

accordingly. The inlet side (termed R_1) comprised a pressurized Falcon tube containing deionized water, connected to the flow sensor via a micro-tube. To stabilize the flow, a high-resistance micro-tube was positioned downstream of the sensor and coupled to a standard tube leading into the microfluidic chip.

On the outlet side (termed R_2), a second Falcon tube filled with the same volume of DI water was left open to atmospheric pressure, with a micro-tube submerged inside. The chip outlet was connected to this tube, forming a single-phase liquid system and maintaining equal hydrostatic conditions across the chip. The horizontal alignment of the microfluidic resistance chip and the tubes helped the pressure standardization of the experiments.

The entire fluidic path was pre-filled with liquid to eliminate air bubbles. After ensuring that the flow was stabilized at 0 ± 0.02 µL/min upon the inlet pressure was reduced to 0 mbar, we confirmed the absence of hydrostatic pressure-driven flow and established baseline conditions. Considering the sensor's calibrated range (0–7 µL/min), we applied inlet pressures between 40 and 600 mbar to ensure measurements fell within this range.

Three experiments were conducted to determine resistance values: (i) ramping the inlet pressure from 40 to 600 mbar over 50 seconds, (ii) repeating the same ramp over 100 seconds, and (iii) applying a constant pressure of 400 mbar and allowing the system to stabilize for 10 minutes. The resistances from the first two experiments were calculated from the slopes of the pressure–flow rate curves (**SI Fig. 3c, d**), while in the third case, resistance was determined by dividing the constant applied pressure (400 mbar) by the corresponding steady-state flow rate.

To quantify the system's intrinsic resistance, a "system chip" was fabricated with a short (10 mm) and wide (800 µm × 800 µm) channel that minimized hydraulic resistance while preserving inlet and outlet geometries. The resistance obtained from this chip was considered the baseline system resistance.

Next, the system chip was replaced with the microfluidic maze of interest. By subtracting the baseline resistance from the total measured resistance, we isolated the resistance contribution of the maze geometry alone. This allowed for a direct comparison with simulation results, which were performed on the maze geometry independent of inlet and outlet effects. For each maze design, resistance values from the three experiments were averaged to obtain the final experimental resistance. The measured values from each experiment are provided in **SI Table 1**. Although the steady-state measurement at 400 mbar was sufficient for comparison, averaging across the three experimental conditions yielded a more robust estimate. Notably, minor variations in calculated resistance were observed when steady flows were measured at 200, 400, and 600 mbar, likely due to subtle internal instabilities of the pressure controller and flow sensor. To mitigate the influence of such fluctuations, we adopted the averaged resistance value as the representative measurement for each maze.

All measurements were conducted using the same flow rate sensor. Testing with different sensors of the same model revealed up to 10% variability, and the sensor output was found to be sensitive to environmental and fluidic conditions. To minimize these effects, the system resistance was re-measured immediately prior to each maze experiment, and all measurements were performed in rapid succession. By combining time-varying and steady-state protocols, we minimized the impact of sensor drift and pressure fluctuations, thereby improving the overall robustness and reliability of the resistance measurements.

The experimentally determined resistance values across all eight maze designs ranged from 0.2 to 7.8 mbar/(µL/min). Simulations were carried out using the actual measured dimensions

of each channel. The corresponding numerical values used in main article **Fig. 2** are provided in **SI Table 1**.

SI Table 1. Experimental resistance measurement results for eight microfluidic maze designs used to validate the simulation method. The values were obtained using the workflow described in **SI Fig. 3**. The ‘system chip’s resistance was separately measured immediately before each resistance chip. Calculation of every single chip’s resistance by comparing to nearly concurrent system chip recordings helped reducing possible time-dependent environmental effects on sensor sensitivity. The “50 s” and “100 s” entries correspond to ramp-up experiments with pressure increasing from 40 to 600 mbar over 50 and 100 seconds, respectively. The “Steady 400 mbar” entry reflects resistance calculated from the steady-state flow rate at a constant 400 mbar inlet pressure. The average of these three values was used as the final resistance measurement for each design.

Design 1

	Design 1 [mbar/(μL/min)]	System [mbar/(μL/min)]	Difference [mbar/(μL/min)]
**50 s**	97.10	96.78	0.32
**100 s**	96.80	96.17	0.63
**Steady 400 mbar**	92.81	92.38	0.43
		**Average**	0.46

Design 2

	Design 2 [mbar/(μL/min)]	System [mbar/(μL/min)]	Difference [mbar/(μL/min)]
**50 s**	97.84	96.37	1.47
**100 s**	98.79	94.88	3.91
**Steady 400 mbar**	94.34	91.53	2.81
		**Average**	2.73

Design 3

	Design 3 [mbar/(μL/min)]	System [mbar/(μL/min)]	Difference [mbar/(μL/min)]
**50 s**	95.52	95.10	0.42
**100 s**	95.68	95.11	0.57
**Steady 400 mbar**	92.81	92.38	0.43
		**Average**	0.47

Design 4

	Design 4 [mbar/(μL/min)]	System [mbar/(μL/min)]	Difference [mbar/(μL/min)]
**50 s**	94.34	94.19	0.15
**100 s**	94.13	94.01	0.12
**Steady 400 mbar**	90.50	90.09	0.41
		**Average**	0.23

Design 5

	Design 5 [mbar/(μL/min)]	System [mbar/(μL/min)]	Difference [mbar/(μL/min)]
**50 s**	96.74	95.94	0.80
**100 s**	96.75	95.12	1.63
**Steady 400 mbar**	93.90	92.81	1.09
		**Average**	1.17

Design 6

	Design 6 [mbar/(μL/min)]	System [mbar/(μL/min)]	Difference [mbar/(μL/min)]
**50 s**	94.20	93.60	0.60
**100 s**	94.43	93.88	0.55
**Steady 400 mbar**	90.91	90.50	0.41
		**Average**	0.52

Design 7

	Design 7 [mbar/(μL/min)]	System [mbar/(μL/min)]	Difference [mbar/(μL/min)]
**50 s**	97.75	95.12	2.63
**100 s**	97.66	94.88	2.78
**Steady 400 mbar**	94.79	91.53	3.25
		**Average**	2.89

Design 8

	Design 8 [mbar/(μL/min)]	System [mbar/(μL/min)]	Difference [mbar/(μL/min)]
**50 s**	100.69	93.40	7.29
**100 s**	100.83	93.66	7.17
**Steady 400 mbar**	100.00	91.12	8.88
		**Average**	7.78

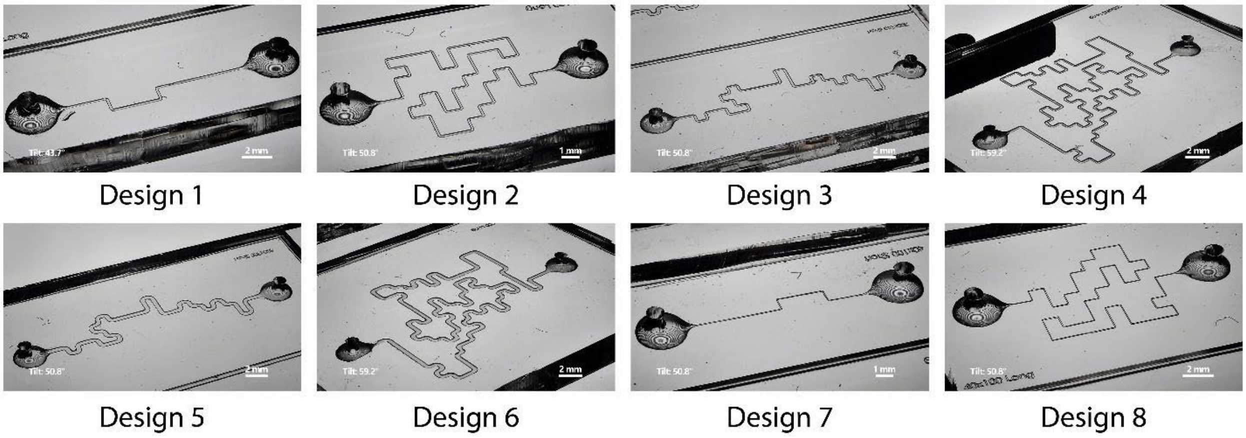

SI Figure 4. Tilted microscope images of the fabricated microfluidic maze designs. The numbering of the designs corresponds to the order presented in **Fig. 2** of the main article. Scale bars and tilt angles vary between images; their respective values are indicated within each image.

SI Table 2. Measured channel cross-sectional dimensions and corresponding resistance values obtained from experiments and simulations.

Design #	Width [µm]	Height [µm]	Experimental Resistance [mbar/(µL/min)]	Simulation Resistance [mbar/(µL/min)]
1	149.6	70.4	0.46	0.61
2	140.7	71.0	2.73	3.10
3	209.7	118.9	0.47	0.36
4	417.9	119.1	1.17	1.37
5	198.8	113.0	0.23	0.13
6	404.2	111.1	0.52	0.52
7	56.0	68.2	2.89	3.36
8	73.9	67.9	7.78	8.85

SI.4. Testing and Classification of Regressor Models

We explored whether utilizing different regressors for specific input conditions could improve predictive performance rather than optimizing a single regressor model. To this end, we initially tested 27 different regression algorithms. The built-in libraries of LazyPredict were used to evaluate the regressors on our dataset. The R-squared values and RMSEs obtained for each regressor are listed in **SI Table 3**. Models in the table was listed by prioritizing (i) the highest adjusted R-squared, (ii) the lowest MAPE, and (iii) the minimum elapsed training time from top to bottom. Five best-performing regressors (Adjusted R-squared above 0.8 and Test MAPE below 0.3%) were selected for further integration into our combined machine learning model.

SI Table 3. R-squared and RMSE results of 27 different regressors.

Regressor	Adjusted R2	R2	Train RMSE	Test RMSE	Train MAPE [%]	Test MAPE [%]	Time [s]
**HistGradientBoosting**	1	1	0.34	0.34	0.02	0.02	0.94
**XGB**	1	1	0.31	0.31	0.02	0.02	0.61
**LGBM**	1	1	0.34	0.34	0.02	0.02	0.42
**Kneighbors**	1	1	0.39	0.57	0.01	0.02	0.65
**GradientBoosting**	1	1	0.52	0.52	0.05	0.05	14.79
**AdaBoost**	0.92	0.92	7.02	7.05	0.46	0.46	11.07
**LassoLarsCV**	0.88	0.88	7.64	7.76	0.47	0.46	0.32
**LassoLarsIC**	0.88	0.88	7.64	7.76	0.47	0.46	0.15
**OrthogonalMatchingPursuitCV**	0.88	0.88	7.64	7.76	0.47	0.46	0.25
**Ridge**	0.88	0.88	7.64	7.76	0.47	0.46	0.16
**BayesianRidge**	0.88	0.88	7.64	7.76	0.47	0.46	0.13
**TransformedTarget**	0.88	0.88	7.64	7.76	0.47	0.46	0.13
**LinearRegression**	0.88	0.88	7.64	7.76	0.47	0.46	0.11
**RidgeCV**	0.88	0.88	7.64	7.76	0.47	0.46	0.32
**SGD**	0.88	0.88	7.53	7.65	0.47	0.47	0.23
**Huber**	0.88	0.88	7.57	7.69	0.46	0.46	0.85
**LinearSVR**	0.88	0.88	7.8	7.92	0.46	0.46	5.32
**RANSAC**	0.86	0.86	6.99	7.11	0.49	0.49	0.24
**Poisson**	0.82	0.82	9.15	9.25	0.69	0.69	0.24
**Gamma**	0.72	0.72	12.12	12.24	0.75	0.76	0.37
**Tweedie**	0.69	0.69	12.5	12.64	0.83	0.83	0.22
**PassiveAggressive**	0.68	0.68	10.51	10.54	0.87	0.87	0.23
**OrthogonalMatchingPursuit**	0.36	0.36	14.18	14.32	1.49	1.49	0.08
**ElasticNet**	0.15	0.15	15.72	15.86	2.28	2.3	0.14
**Lasso**	0	0	16.19	16.33	2.77	2.79	0.09
**LassoLars**	0	0	16.19	16.33	2.77	2.79	0.09
**Dummy**	0	0	16.19	16.33	2.77	2.79	0.07

After selecting the five best-performing models, we investigated whether they approached the data differently. XGB, LGBM and GradientBoosting regressors provides built-in functions for calculating the importance of input features, which we utilized for the selected models. In this analysis, feature importance was determined based on the percentage effect of each input parameter on the predicted resistance. As shown in **SI Fig. 5**, although all models assigned the superior importance to channel length, width, and height—consistent with physical expectations—the relative weighting of these features varied across different regressors. This observation confirmed that some regressors perform better for certain design conditions, while others outperform in different configurations. Based on this insight, we introduced a classification strategy for regressors according to their performance profiles. This allowed our combined model to dynamically select the most suitable regressor for predicting the resistance of a given maze design, ensuring optimal accuracy across varying microfluidic architectures.

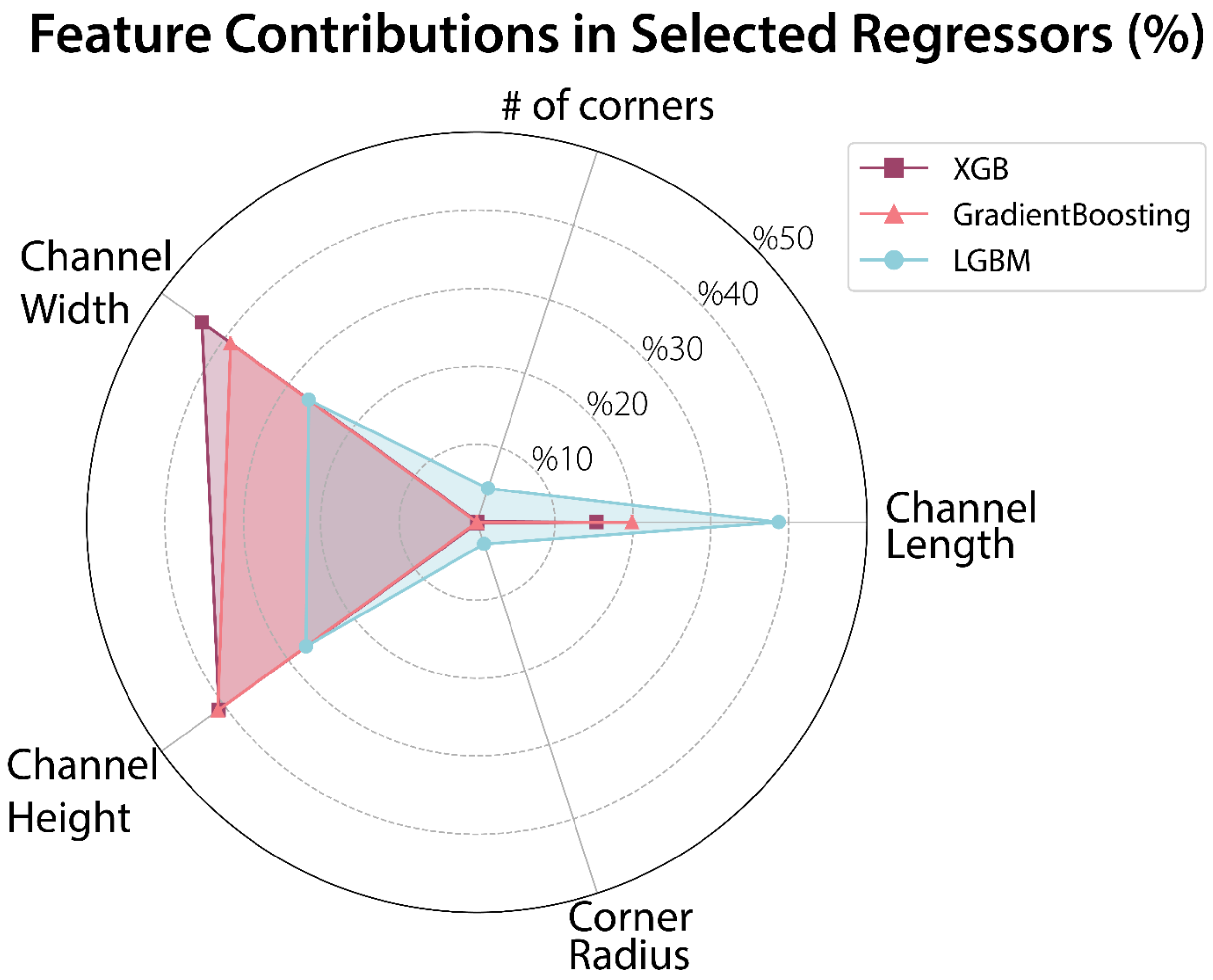

SI Figure 5. Feature importance profiles for selected regressors used in resistance prediction. The percentage importance of each input feature was calculated using the built-in feature importance functions of the XGBoost, GradientBoosting, and LightGBM regressors from the LazyPredict library. The observed variation in feature rankings across models highlights that different regressors prioritize different aspects of the input data, underscoring the benefit of using a regressor-specific classification approach in the ensemble framework.

To further assess the generalization performance of the selected regressors, we examined how their error trends evolved with increasing training data. **SI Fig. 6** presents plots of training data percentage versus MSE for each of the five models. Across all regressors, we observed parallel and consistent decreases in MSE for both training and test sets as the amount of training data increased. This parallel behavior indicates that none of the models were overfitting to the training data, as test error followed a similar trend to training error without sudden divergence. Our goal with this analysis was to verify the stability and scalability of each regressor when exposed to different volumes of data. The observed alignment in learning curves supports the conclusion that all selected models exhibit robust generalization performance and are suitable for integration into our adaptive prediction framework.

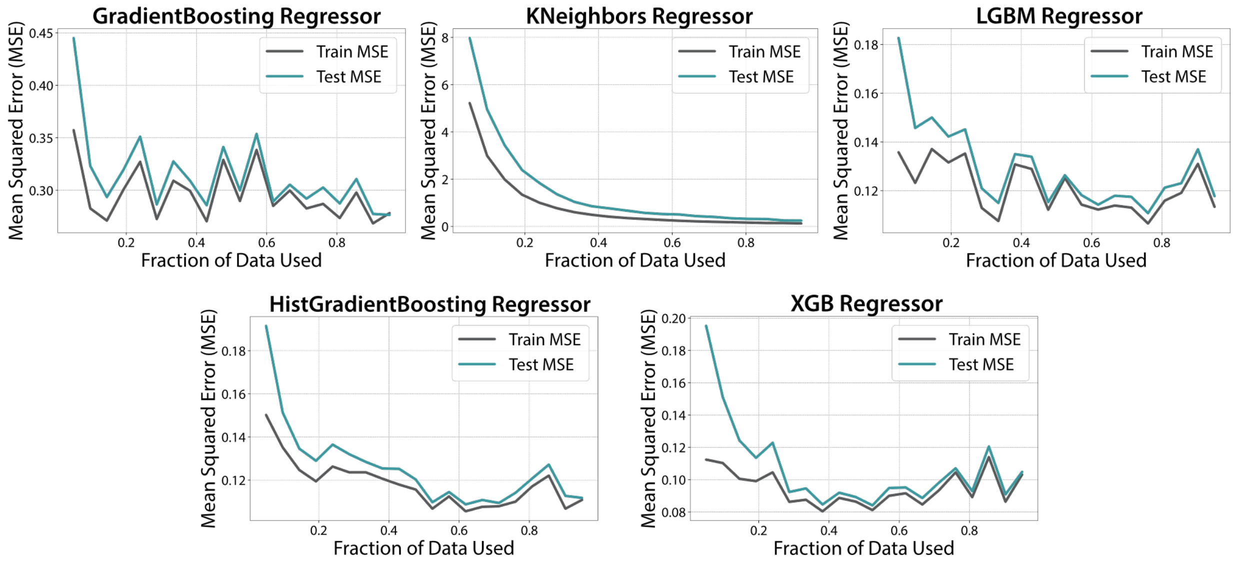

SI Figure 6. Mean squared error (MSE) versus training data percentage for the five best-performing regressors. For each model, both training and test set errors are plotted to evaluate generalization performance. All regressors display parallel trends between training and test errors as the amount of training data increases, indicating consistent learning behavior and the absence of overfitting. This supports the robustness of the selected models for scalable resistance prediction across diverse microfluidic maze geometries.

The selection of the classifier algorithm for assigning regressors was carried out using a straightforward performance-based approach. We evaluated 20 different classification models available in the LazyPredict library, as listed in **SI Table 4**. Each classifier was assessed using the library's built-in accuracy metric, which reflects the proportion of correct predictions made by the model on the test set. Models were then ranked solely based on this accuracy score. Among all tested classifiers, the Bagging Classifier consistently yielded the highest accuracy and was therefore selected for use in our framework to assign the most appropriate regressor to each input condition. Since this classifier achieved near-perfect performance in our application, we did not take additional parameters such as training time or complexity into account during the selection process. However, we note that in other contexts or datasets where classification is more challenging, these parameters might play a more significant role and should be considered based on the specific demands of the application.

SI Table 4. Performance metrics of 20 classifier models tested for regressor assignment. Each classifier was evaluated using LazyPredict's built-in functions, reporting accuracy, balanced accuracy, and time taken for training and prediction.

Classifier	Accuracy	Balanced Accuracy	Time Taken [s]
**Bagging**	0.724	0.702	5.552
**DecisionTree**	0.701	0.679	0.914
**RandomForest**	0.674	0.633	25.240
**ExtraTrees**	0.667	0.631	25.349
**ExtraTree**	0.524	0.490	0.651
**Kneighbors**	0.458	0.404	3.917
**LGBM**	0.481	0.376	1.824
**NearestCentroid**	0.278	0.286	0.923
**AdaBoost**	0.383	0.280	18.147
**QuadraticDiscriminantAnalysis**	0.369	0.267	0.846
**GaussianNB**	0.362	0.262	0.749
**LinearDiscriminantAnalysis**	0.366	0.261	1.423
**LogisticRegression**	0.366	0.261	2.298
**CalibratedClassifierCV**	0.366	0.261	4.907
**RidgeClassifier**	0.364	0.259	0.666
**RidgeClassifierCV**	0.364	0.259	0.856
**LinearSVC**	0.364	0.259	2.048
**SGDClassifier**	0.343	0.253	1.047
**BernoulliNB**	0.350	0.250	0.668
**Perceptron**	0.277	0.223	1.903

The comparative performance of individual regressors and our combined model is presented in **SI Fig. 6**. Notably, the test RMSE of the combined model outperformed the best standalone regressor—XGBoost—by approximately 40%. Similarly, the test MAPE was reduced by 43% compared to the top-performing single model in that metric, which was the K-Neighbors Regressor. These results clearly demonstrate that our strategy of leveraging multiple regressors through a classification-guided framework significantly enhances predictive accuracy. This improvement is particularly critical in our application, where the underlying physical phenomena—namely fluidic resistance in microchannels—require extremely sensitive modeling of geometric parameters such as channel length, width, and height. Minor variations in these dimensions can lead to substantial shifts in resistance, making precision essential. To address this, we implemented a hybrid regressor model that dynamically selects the most appropriate estimator based on the input geometry, thereby improving overall robustness and sensitivity in the prediction of microfluidic resistance.

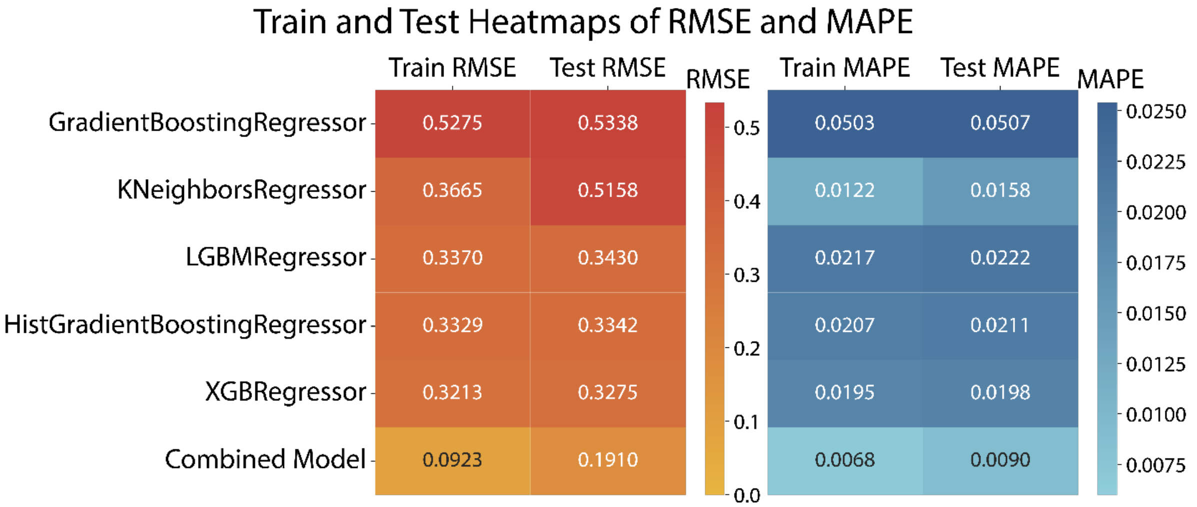

SI Figure 7. Comparison of prediction performance among top regressors and the μFG combined model. Root Mean Square Error (RMSE) and Mean Absolute Percentage Error (MAPE) are shown for the five best-performing individual regressors, alongside the combined model implemented in μFG. The ensemble approach demonstrates superior accuracy across both metrics, highlighting the benefit of regressor-specific assignment over single-model strategies.

SI.5. 3D Model Creation in μFG App

The μFG interface integrates a mathematical solver and a machine learning-based generative algorithm to design circuits with specified resistances. The resulting maze-like channel pathways are stored as coordinate lists representing the centerlines of each microfluidic segment.

Channel components—including inlets, outlets, and junctions—were pre-modeled in CAD software and saved as individual .stl files (**SI Fig. 8**). These components were designed with variable heights ranging from 50 μm to 200 μm, allowing seamless integration with any channel thickness specified during the design process. The Python library trimesh was used to handle 3D mesh operations. During model generation, *.stl* components were imported and aligned with the computed coordinate paths to define the final channel geometries, with cross-sectional dimensions assigned programmatically.

All maze designs were confined within a standardized 10 × 10 mm area, and inlet/outlet locations within this area were fixed by default. This allowed consistent and modular alignment of maze outputs with other components such as pre-designed junctions, reservoirs, or downstream modules. These standardized attachment points ensured compatibility between generated designs and multi-module microfluidic architectures.

All pre-modeled 3D components are available for download through our GitHub repository at [https://github.com/dxbiotech/Microfluidics-Resistance-ML]. The standalone μFG application was developed using the tkinter Python GUI library, and the executable software along with a detailed user guide can be accessed at our lab website: [https://dxbiotech.ku.edu.tr/guidelines4829438573810849/guideline-microfluidic-generative-design-app-user-guideline/].

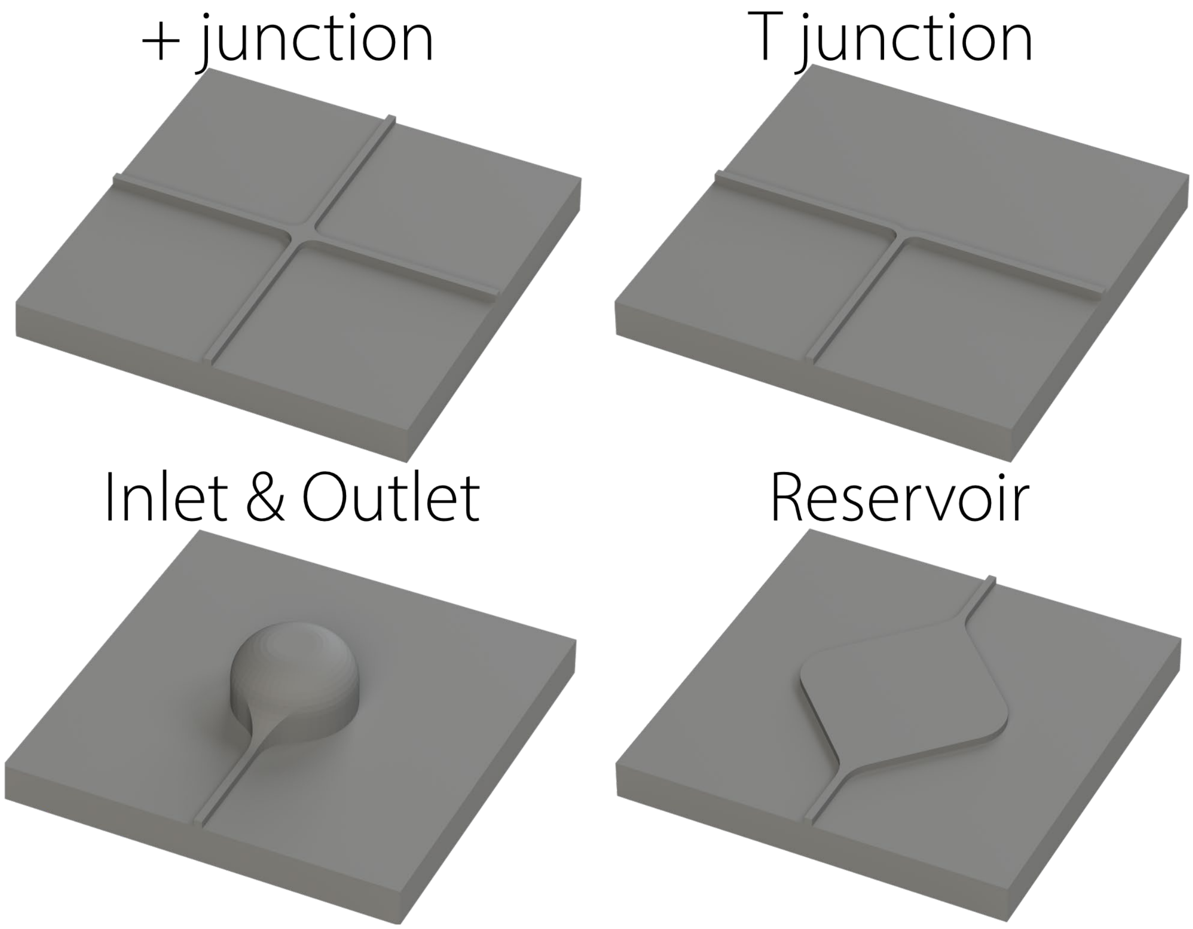

SI Figure 8. CAD renders of the pre-built design elements used in μFG. All structures were constructed on a standardized 10 × 10 mm base, with channel connections positioned at the midpoint of each edge to allow seamless integration with resistance-tailored mazes. Channel heights for these components were made 10 μm increments, ranging from 50 to 100 μm. The effect of height on overall resistance is reduced above 100 μm. Thus, 150 and 200 μm variations were also included to help with systems with low resistances. These parts are embedded within the μFG software and managed via the trimesh library, which uses coordinate paths generated by the ML-based algorithm. If the user selects the 'Add Base' option within the interface, a base layer is automatically added beneath the maze design, and outer walls are generated programmatically. If this option is deselected, a negative extrude operation is applied to remove base structures across the design, yielding a channel-only 3D model suitable for alternative fabrication workflows.

SI.6. Evaluation of μFG-Generated Resistances

To evaluate the ability of μFluidicGenius (μFG) to generate channels with specific hydraulic resistances, we conducted a series of tests using the software's basic interface. Each test involved connecting a single inlet and outlet through a straight channel and specifying an inlet pressure and target flow rate. Eight distinct resistance values were targeted (0.5, 1, 2, 4, 8, 10, 20, and 40 mbar/(μL/min)), as summarized in **SI Table 5**. For example, to achieve a resistance of 4 mbar/(μL/min), an inlet pressure of 20 mbar and a flow rate of 5 μL/min were entered into the software. All flow rates were selected to remain below 7 μL/min to ensure compatibility with our pressure sensors and to maintain low Reynolds number conditions consistent with laminar flow.

Fabricated devices exhibited dimensional deviations from the original μFG-generated designs, as listed in **SI Table 5**. These discrepancies primarily arose from unpredictable variations introduced during the 3D printing process, particularly due to the mold's position on the printer's build plate. Such deviations had a more pronounced impact on channels with smaller cross-sectional dimensions, where even minor manufacturing imprecision significantly affected the resulting resistance. A comparison between the μFG target values and

experimentally measured resistances revealed an average deviation of approximately 28%, with some individual errors reaching up to 8 mbar/(µL/min).

To better understand and account for these deviations, we used µFG's machine learning-based resistance prediction model. By inputting the actual measured dimensions of the fabricated devices along with the corresponding design layouts, the model produced updated resistance predictions. These ML-based predictions reduced the average deviation between expected and measured resistances to approximately 12%, demonstrating the value of incorporating fabrication-aware features into the modeling pipeline. In parallel, COMSOL simulations were performed using the same measured geometries to generate a separate set of resistance predictions, also included in **SI Table 5**. The resistance values presented in **Fig. 3d** of the main text are based on these measurement-informed predictions.

Experimental resistance measurements and simulation procedures were carried out as described in **Sections SI.3** and **SI.1**, respectively.

SI Table 5. Details of µFG-generated resistance elements corresponding to Fig. 3d in the main article. Design w & h refer to the channel width and height output by µFG, while Meas. w & h indicate the actual dimensions measured from the fabricated channels. The experimental resistance values (R) were calculated using the method described in SI.3. Both the µFG predictions and COMSOL simulations were based on the measured channel dimensions to account for fabrication-induced variability..

Target R [mbar/(µL /min)]	Design w [µm]	Design h [µm]	Meas. w [µm]	Meas. h [µm]	Experimental R [mbar/(µL/min)]	µFG R Prediction Meas. w&h [mbar/(µL/min)]	COMSOL R Calculation Meas. w&h [mbar/(µL/min)]
0.5	200	80	225.2	72.7	0.8	0.7	0.6
1	70	150	78.6	142.1	0.8	0.7	0.8
2	100	70	120.3	70.4	1.9	1.9	1.5
4	80	60	107.5	48.5	3.7	4.2	4.4
8	60	50	88.2	50.5	5.0	6.0	5.2
10	60	50	73.4	48.1	13.9	12.1	11.8
20	50	50	69.0	48.1	27.9	22.9	25.1
40	50	50	71.6	47.6	47.1	43.6	47.4

SI.7. Evaluation of uFG Generated Microfluidic Circuits

Unlike the experiments conducted for measuring the resistance of single-inlet, single-outlet channels, the flow rate validation of multi-outlet circuits required simultaneous monitoring of multiple fluidic paths. To achieve this, three flow rate sensors were used concurrently (**SI Fig. 9**). Channel 1 of the OB1 pressure controller was connected to an MFS3 sensor (ranged ±80 µL/min), which was arranged in series with the inlet tubing containing a microfluidic resistance. This sensor enabled accurate measurement of higher flow rates delivered into the system. Channels 2 and 3 were each connected to MFS2 sensors (ranged ±7 µL/min), which provided higher sensitivity and were used to monitor flow rates at the outlets of the circuits.

All experiments were performed in a fully water-filled system to eliminate trapped air bubbles in the tubing or channels. A microfluidic resistance placed upstream of the inlet served to stabilize flow and introduce a pressure threshold. Approximately 100 mbar of pressure was required to initiate flow through the system when no additional chip resistance was present. Accordingly, all experimental inlet pressures were set above this threshold. After applying the desired pressure during each experiment, the system was allowed to stabilize until steady-state flow was reached. Flow rate recordings were then initiated to generate the data presented in **SI Fig. 11**.

The dimensional consistency of the fabricated microfluidic chips was verified before the experiments by measuring the channel widths and heights across multiple designs. For the two-outlet design, µFG specified a channel height of 100 µm and a width of 150 µm. The measured heights were consistently ~110 µm, while the widths across all three branches deviated by no more than ±4 µm from the target value. In the three-outlet design with 100 µm height and 70 µm width, the measured heights were again consistent at ~109 µm, and the widths varied within ±8 µm of the intended dimensions. For another three-outlet design targeting 150 µm width and 80 µm height, the channel widths deviated up to ±10 µm, while the heights measured consistently at ~74 µm—6 µm below the design specification. Overall, dimensional deviations were small and consistent across each design, which contributed to achieving the user-defined flow rate distributions with high fidelity. These results confirm that the fabrication constraints embedded within µFG's mathematical modeling framework effectively ensured manufacturable designs, as all chips were successfully 3D printed and reproduced target flow conditions with reliable accuracy.

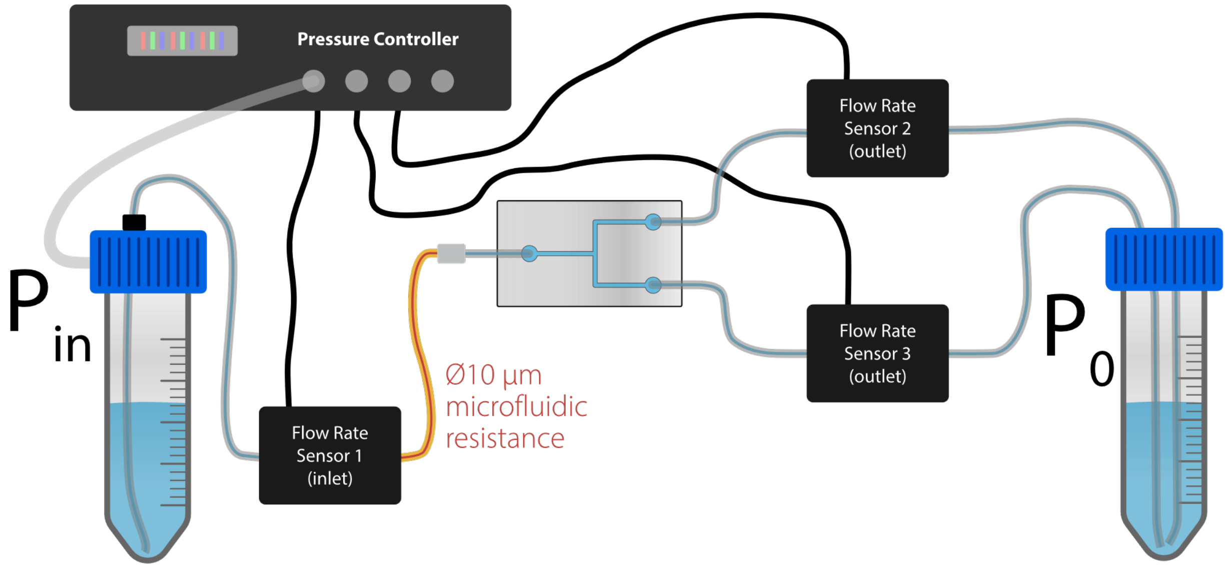

SI Figure 9. Experimental setup for measuring µFG-generated microfluidic circuits with multiple outlets. Unlike the single-resistance measurement setup, this configuration employed three flow sensors operating simultaneously. Flow Sensor 1 (0–80 µL/min range) was consistently placed at the inlet to monitor total input flow. The remaining two sensors were connected to the outlets of the tested chips. All flow data were recorded concurrently to ensure consistent inlet conditions and accurate distribution measurements. For the two-outlet chip (Design 1), each outlet was connected to a flow sensor, as depicted. For the three-outlet chip (Design 2), one outlet was continuously monitored by Sensor 2, while Sensor 3 was alternated between the remaining two outlets in successive measurements. In each case, the outlet not connected to a sensor was routed to a disposal line. Measurements were only recorded after flow stabilization, and Sensor 2's readings were kept within ±0.2 µL/min across all configurations to ensure comparability. A similar approach was used for the four-outlet chip (Design 3): Sensor 2 monitored one outlet continuously, while Sensor 3 was sequentially connected to the remaining three outlets, with the others routed to disposal. This modular setup enabled robust and reproducible validation of µFG-generated flow-splitting circuits within the operational range of the available equipment.

In the two-outlet circuit configuration, the experimental setup shown in **SI Fig. 9** was used. An initial inlet pressure of ~110 mbar was applied. Early measurements showed outlet flow rates close to the uFG-predicted values of ~3.9 µL/min and ~1.0 µL/min. However, during stabilization, flow through outlet 2 gradually decreased and eventually ceased. The inlet pressure was then increased to ~120 mbar, resulting in stable flow rates of ~5.7 µL/min and ~1.5 µL/min through outlets 1 and 2, respectively. These readings were recorded for 5 minutes and are presented in **SI Fig. 11a**.

A different arrangement of sensors was used to measure flow rates through three outlets in the next circuit configuration. The goal in this design was to distribute an inflow of ~12 µL/min

into ~6, ~4, and ~2 µL/min through outlets 1, 2, and 3, respectively. Two sensors were employed sequentially: initially, sensors were connected to outlets 1 and 2, while outlet 3 was routed directly to the waste falcon tube. After stabilization, flow rates of ~6.7 µL/min and ~4.0 µL/min were recorded through outlets 1 and 2. Following a 300-second recording period, the second sensor was removed from outlet 2 and connected to outlet 3, while outlet 2 was connected to waste. Outlet 1 remained connected to the sensor and maintained a flow of ~6.7 µL/min, while outlet 3 was measured ~1.9 µL/min after reaching steady flow regime. This method allowed relative flow measurements under consistent conditions, leveraging the three available channels of the OB1 pressure controller. Inlet pressure was maintained at ~150 mbar throughout the experiment. Corresponding flow profiles are provided in **SI Fig. 11b**.

In the four-outlet circuit, the uFG design targeted flow rates of ~6, ~5, ~4, and ~3 µL/min through outlets 1, 2, 3, and 4, respectively, driven by an inflow of ~18 µL/min. An inlet pressure of ~175 mbar was applied. One flow rate sensor was continuously connected to outlet 1, while the second was sequentially connected to outlets 2, 3, and 4. The two outlets not being recorded were routed directly to waste. Measurements indicated that flow through outlet 1 was consistently maintained at ~6 µL/min. Flow rates at the remaining outlets were measured as ~3.9 µL/min (outlet 2), ~4.9 µL/min (outlet 3), and ~3.0 µL/min (outlet 4). Corresponding flow profiles are provided in **SI Fig. 11c**.

In all experiments, flow rate recordings were collected over 5-minute intervals after steady-state conditions were reached. Averaging these recordings yielded stable and reproducible measurements. During each experiment, the recorded flow rates deviated by no more than ±0.3 µL/min from the specified average values determined for each outlet. The instantaneous peaks observed in the time series plots were caused by unavoidable measurement errors inherent to the flow sensors. Due to their operating principles, these sensors can occasionally register minuscule extremes that do not reflect actual flow changes. While the use of a multi-channel flow reader with additional ports would have improved measurement efficiency, the current setup was optimized to its full capacity. Repeated trials showed consistent results, confirming the reliability of the measured steady-state flow distributions.

Additionally, these 3 circuit designs were simulated manually in COMSOL Multiphysics and corresponding flow profiles are shown in **SI Fig. 10**.

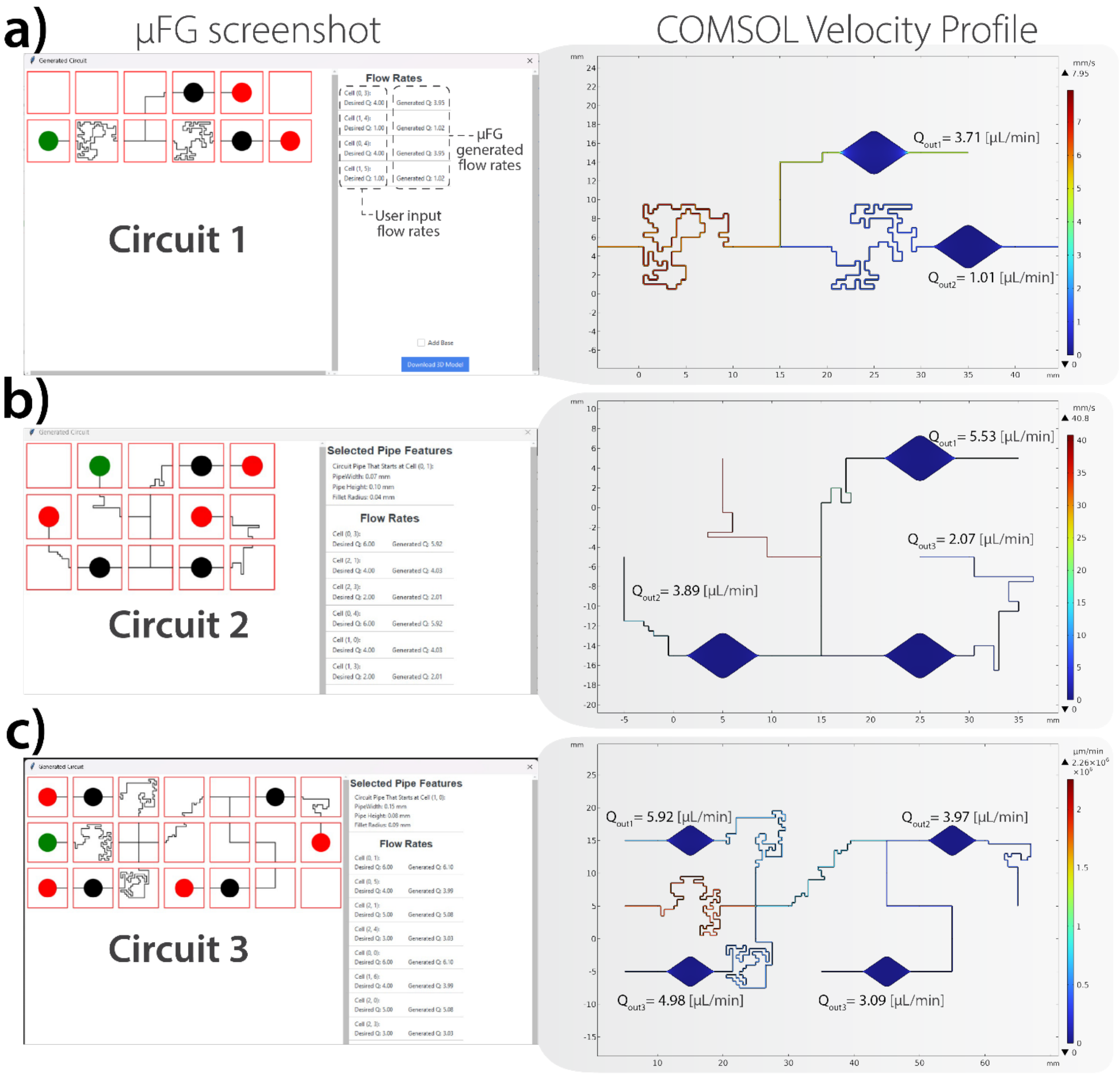

SI Figure 10. Output screenshots of µFG-generated microfluidic circuit designs alongside their corresponding COMSOL-simulated velocity profiles. Panels (**a**), (**b**), and (**c**) respectively display Circuit 1, Circuit 2, and Circuit 3. Each design was created using the µFG interface based on user-defined flow constraints, and subsequently imported into COMSOL Multiphysics for fluid dynamics simulation. The velocity distributions illustrate the flow behavior across the full circuit layout, confirming that the µFG-generated geometries support consistent laminar flow under the specified inlet conditions.

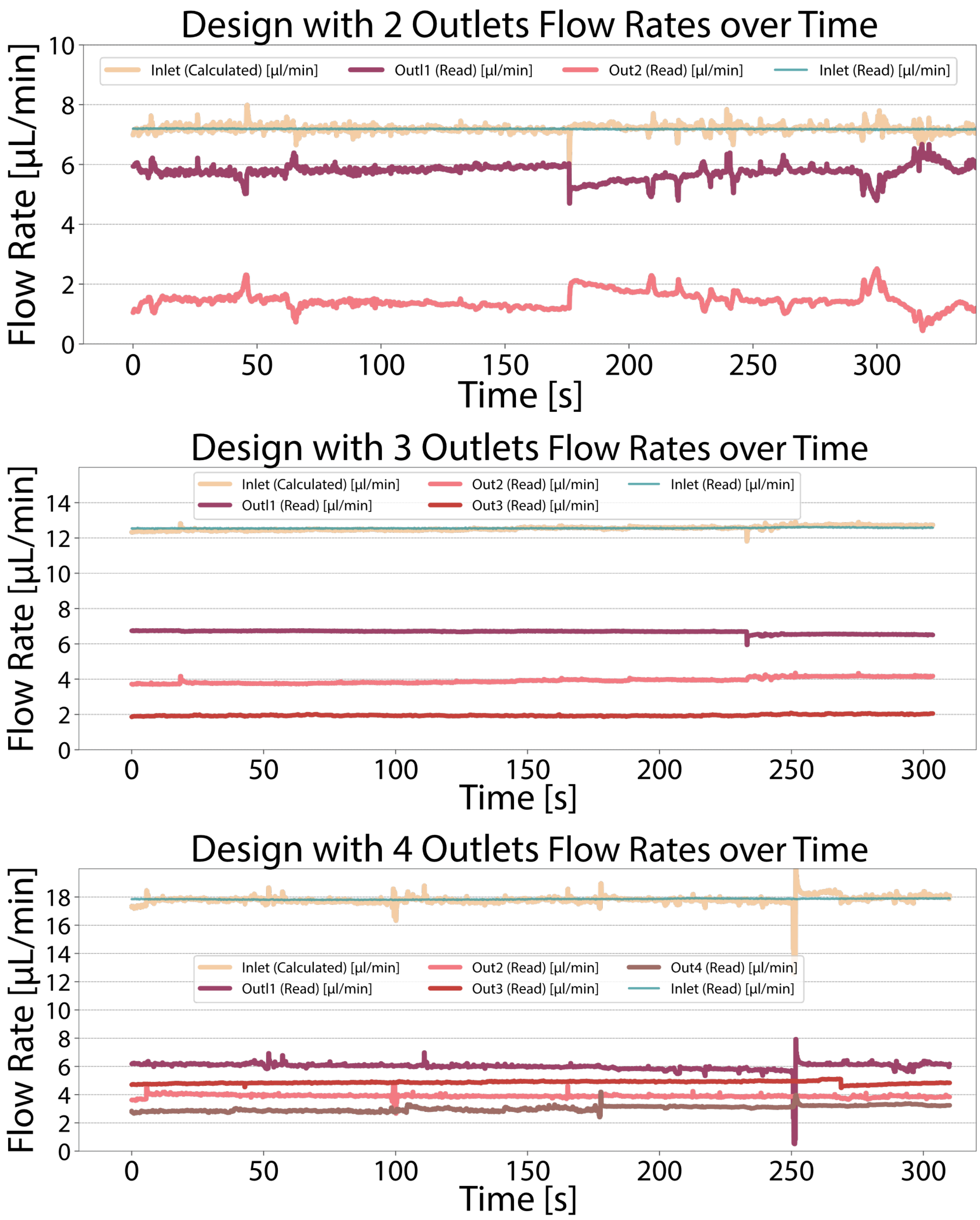

SI Figure 11. Real-time flow rate measurements in µFG-generated microfluidic circuits with one inlet and multiple outlets. The blue lines labeled 'Inlet (Read)' show the real-time readings from Sensor 1 (see **SI Fig. 9**), which continuously monitored the total inlet flow. Readings from Sensors 2 and 3 were used to quantify outlet flow rates, with outlet numbering consistent with the schematic in **main article Fig. 4d**. The yellow lines labeled 'Inlet (Calculated)' represent the sum of outlet flow rates, used to verify mass balance and confirm consistency between measured inlet and outlet values. For the three- and four-outlet designs, flow through Outlets 2, 3, and 4 was each recorded once, while Outlet 1 was recorded three times—once during each configuration where Sensor 3 was connected to a different outlet. This also resulted in three corresponding inlet flow recordings (Sensor 1), one per configuration. Analysis showed that the flow rate measurements for both Outlet 1 and the inlet were highly consistent across all recordings, with variation below ±0.2 µL/min. For simplicity and consistency, we selected the dataset in which Outlet 1 and the inlet were recorded simultaneously with Outlet 2. The time-series traces shown

in the plots reflect this configuration. The average flow values derived from these traces were used to generate the bar plots in **main article Fig. 4d**.

SI.8. Generation of Microphysiological Systems with μFG

Eddington et al.'s microphysiological system (MPS) platforms [62] were reported in three configurations: "4-way," "7-way," and "10-way." In each design, the channels were interconnected to mimic inter-organ circulation, and individual flow rates were controlled through independent pneumatic pumps. The "4-way" configuration contained channels representing liver, gut, endothelial, and lung compartments, with respective target flow distributions of 30%, 50%, 10%, and 10% of the total inflow rate. The "7-way" platform incorporated liver, pancreas, gut, endothelial, lung, heart, and brain compartments with respective target flow distributions of 13%, 2%, 38%, 7%, 5%, 9%, and 26% of the total perfusion rate. Likewise, the "10-way" configuration extended the network to include additional organ compartments—muscle, skin, and kidney—resulting in the following partition: liver 8%, pancreas 1.5%, gut 18%, lung 3%, heart 5%, muscle 20%, brain 14%, endothelium 1.5%, skin 6%, and kidney 23%.

Each of these target flow distributions was entered into μFG as user-defined outlet flow ratios, and the software automatically generated corresponding microfluidic circuits that reproduced the desired partitioning (**SI Table 6**). Generated flow distributions closely reproduced the target partitioning ratios defined in the original MPS systems. For the 4-way design (liver–gut–endo–lung), μFG outputs of 31.7%, 47.4%, 10.1%, and 10.1% corresponded to the required 30%, 50%, 10%, and 10%, respectively, yielding a correlation coefficient of $R^2 = 0.997$. For the 7-way configuration (liver–pancreas–gut–endo–lung–heart–brain), target and generated flow percentages were nearly identical, with $R^2 = 0.999$. Similarly, for the 10-way platform, μFG reproduced the complex flow distribution across ten organ compartments with minimal deviation ($R^2 = 0.999$).

The current version of μFG supports configurations with a single inlet and multiple outlets, enabling systematic generation of parallel microchannel networks. However, it is presently limited in handling channel merging events along the flow path. In other words, while the software can represent crosstalk between serially connected reservoirs, two distinct channels cannot yet merge into a single downstream branch. This constraint arises from the underlying mathematical model, which currently assumes a tree-like topology without looped or converging flow paths. Future versions of μFG will incorporate updated circuit-solvers to include such merging and feedback connections, extending its applicability to more complex architectures like those where pancreatic and intestinal flows join the hepatic branch.

Another distinction from Eddington et al.'s MPS platforms is that the μFG-generated circuits do not recirculate the fluid. Instead, each outlet continuously receives fresh inflow, maintaining steady perfusion rather than cyclic exchange. Despite this difference, the generated designs successfully reproduced the relative flow profiles of the original MPS systems, effectively simulating physiologically relevant flow conditions within a simplified, open-flow format.

SI Table 6. Comparison of target and μFG-generated flow distributions for multi-organ microphysiological systems (MPSs). The table lists the intended outlet flow percentages for 4-, 7-, and 10-way configurations reported by Eddington et al. and the corresponding values autonomously generated by μFG. The resulting distributions show near-perfect correlation between target and generated values ($R^2 = 0.991$ for 4-way, 0.9999 for 7-way, and 0.9997 for 10-way systems), confirming the model's ability to accurately reproduce complex physiological flow partitioning.

System	Outlet	Target (%)	μFG-generated (%)
4-way	Liver	30	31.7
	Gut	50	47.4
	Endo	10	10.1
	Lung	10	10.1
7-way	Liver	13	12.99
	Pancreas	2	1.97
	Gut	38	37.85
	Endo	7	6.93
	Lung	5	4.97
	Heart	9	8.92
	Brain	26	26.09

System	Outlet	Target (%)	μFG-generated (%)
10-way	Liver	8	7.91
	Pancreas	1.5	1.5
	Gut	18	17.85
	Lung	3	3.15
	Heart	5	5.02
	Muscle	20	19.73
	Brain	14	14
	Endo	1.5	1.52
	Skin	6	5.97
	Kidney	23	23.17

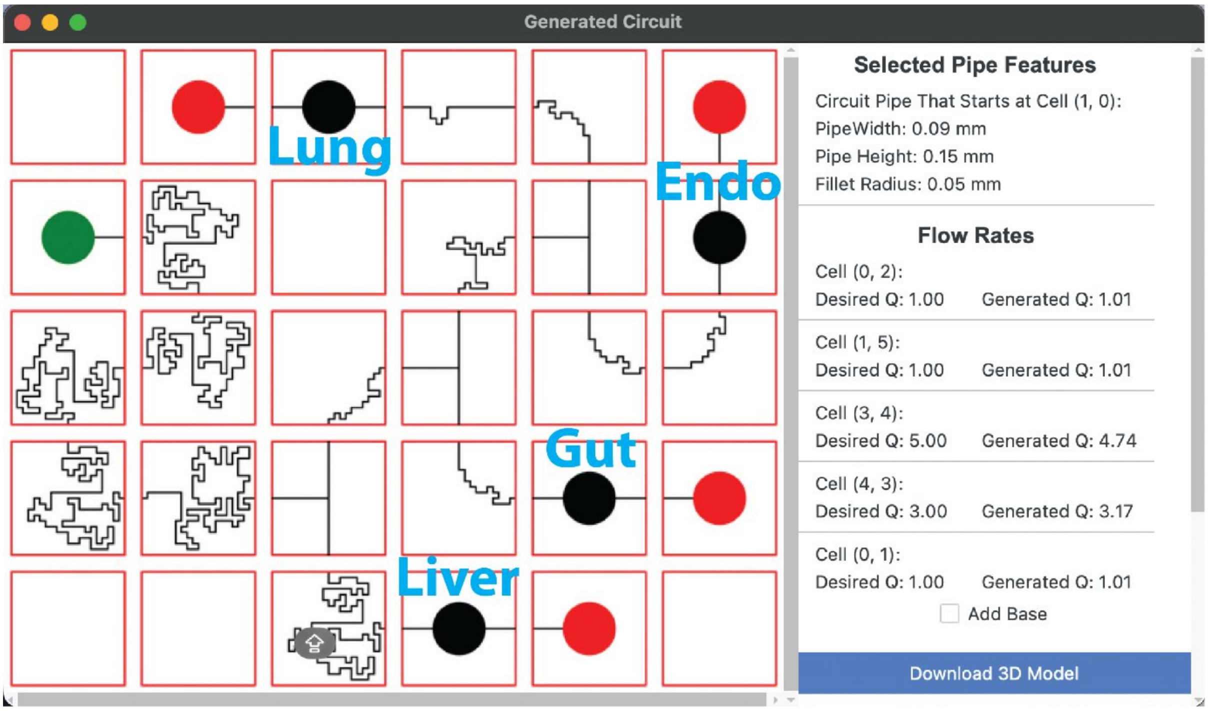

SI Figure 12. μFG-generated microfluidic circuit reproducing the 4-way microphysiological system (MPS) flow distribution. The interface displays the generated cell network (left) and the corresponding flow performance summary (right). Each cell represents a 10 mm × 10 mm maze unit automatically designed to meet the user-defined flow targets. Inlet and outlet positions are indicated by green and red circles, respectively, while black circles denote culture reservoirs. Each reservoir is additionally labeled in blue to indicate the corresponding tissue compartment receiving the specified flow rate. The target flow partition of 3:5:1:1 (liver, gut, endothelium, and lung channels) was accurately reproduced, with μFG generating 3.17, 4.74, 1.01, and 1.01 μL min^{-1}, respectively.

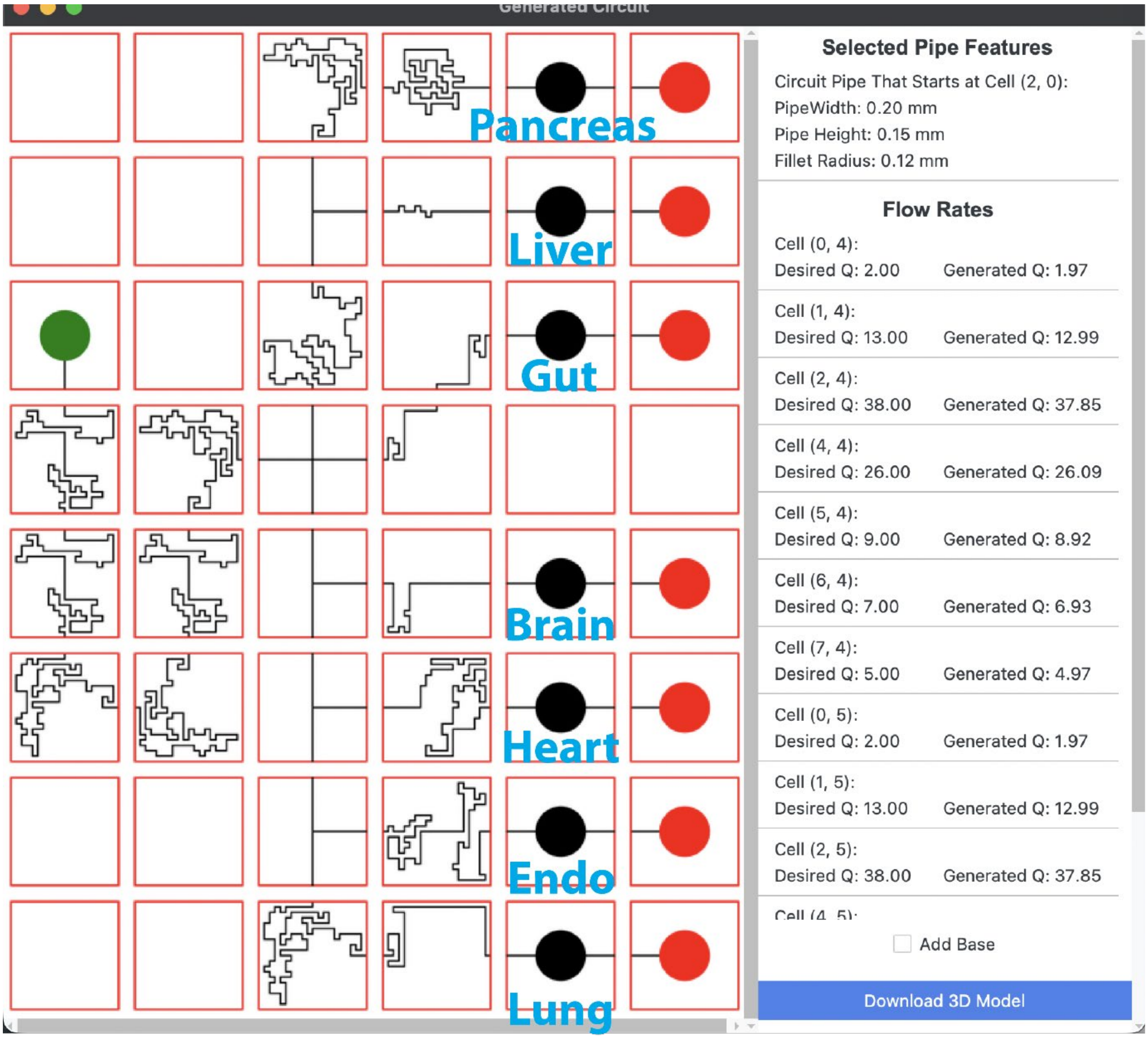

SI Figure 13. μFG-generated microfluidic circuit reproducing the 7-way microphysiological system (MPS) flow distribution. The generated cell network (left) and corresponding flow performance (right) are shown. Each 10 mm × 10 mm maze unit was automatically designed to achieve the specified outlet flow targets. The inlet (green circle) distributes the total inflow among seven outlet channels (red circles), each connected to culture reservoirs (black circles) representing pancreas, liver, gut, endothelium, lung, heart, and brain compartments (labeled in blue). The target flow distribution of 2:13:38:7:5:9:26 (%) was accurately reproduced by μFG, yielding 1.97, 12.99, 37.85, 6.93, 4.97, 8.92, and 26.09 μL min^{-1}, respectively.

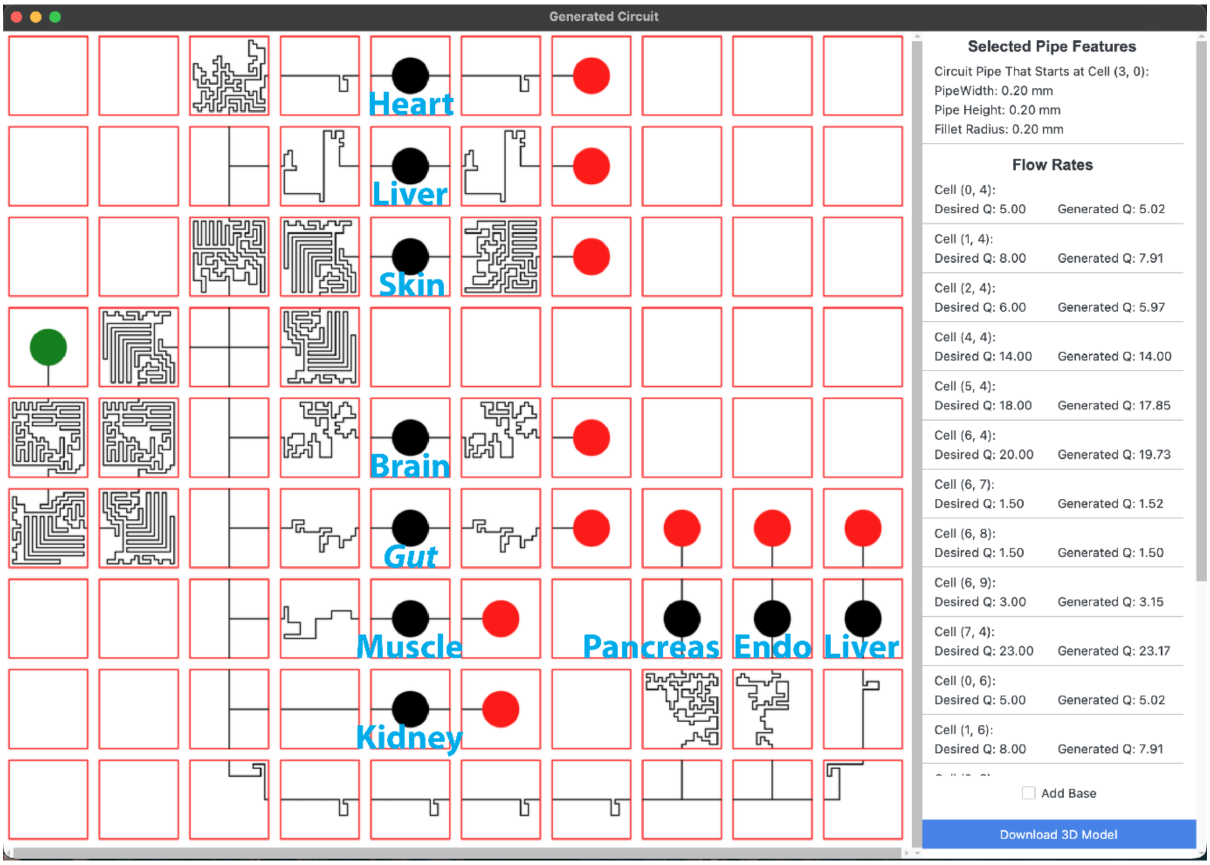

SI Figure 14. μFG-generated microfluidic circuit reproducing the 10-way microphysiological system (MPS) flow distribution. The interface shows the generated circuit layout (left) and the corresponding flow performance data (right). Each cell represents a 10 mm × 10 mm maze unit designed to meet user-specified flow ratios. The inlet (green circle) divides the total inflow among ten outlets (red circles), each linked to a culture reservoir (black circle) representing liver, pancreas, gut, lung, heart, muscle, brain, endothelium, skin, and kidney compartments (labeled in blue). The target flow partition of 8:1.5:18:3:5:20:14:1.5:6:23 (%) was successfully reproduced, with μFG generating 7.91, 1.50, 17.85, 3.15, 5.02, 19.73, 14.00, 1.52, 5.97, and 23.17 μL min^{-1} for the respective outlets.

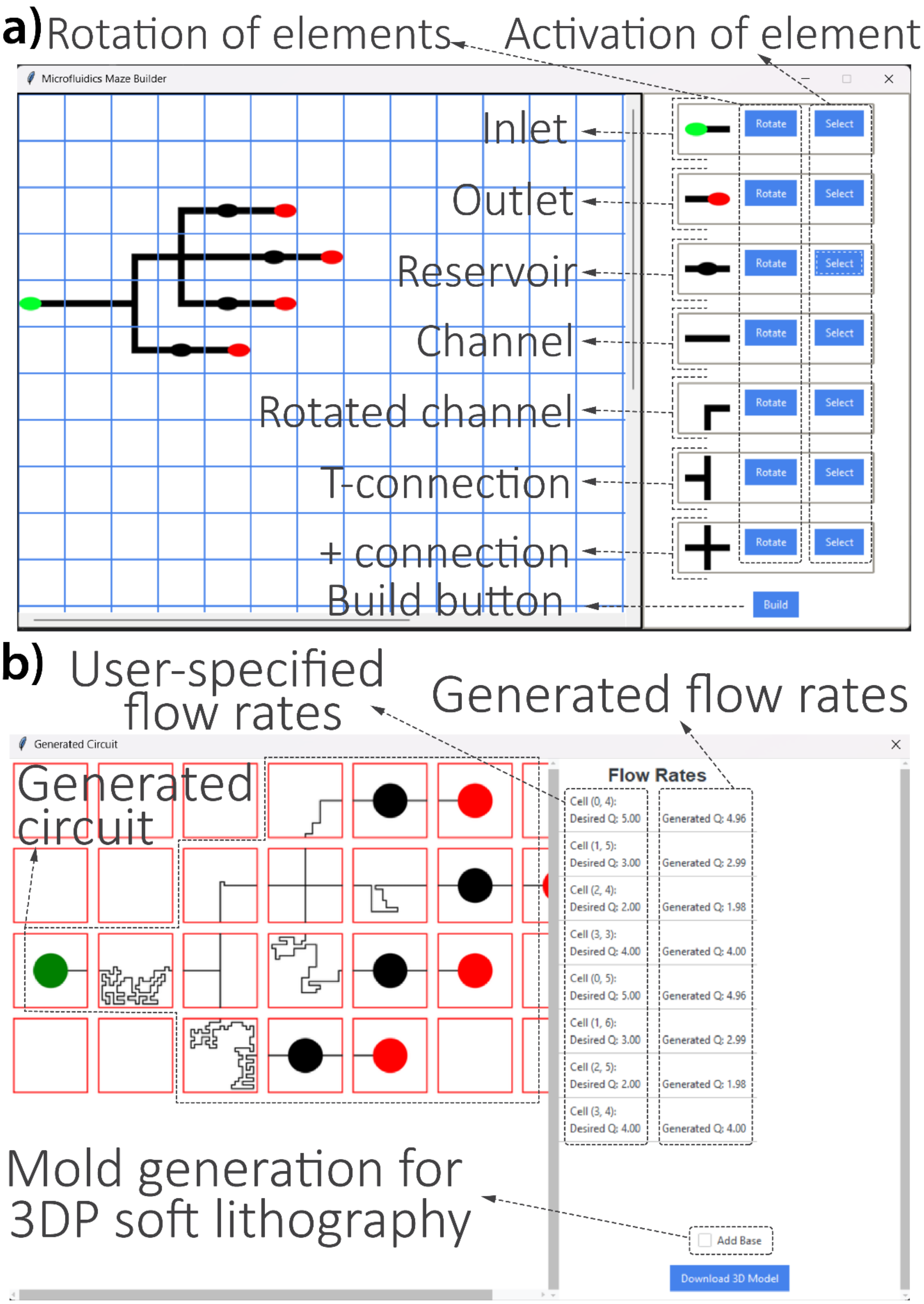

SI Figure 15. Screenshots of the μFG software. (**a**) Shows the interface as it lets user to specify channel patterns. User can activate a channel section by clicking it's 'Select' button and then place it to any square in the matrix. The 'Build' button starts the computation process: the user is asked to enter an inlet pressure and desired flow rates through each reservoir and outlet. If the entered values obey the conservation of mass, the software runs and designs the channel. (**b**) After the calculation, the representative layout of the generated circuit is shown. On the right, the software lists the calculated flow rate (performance) of the freshly designed circuit. The .stl file of the generated circuit is downloaded through the 'Download 3D Model' button. If the 'Add Base' is checked, the downloaded file has shape of a mold that can be used in soft lithography.
